# CAFs secreted exosomes promote metastasis and chemotherapy resistance by enhancing cell stemness and epithelial-mesenchymal transition in colorectal cancer

**DOI:** 10.1186/s12943-019-1019-x

**Published:** 2019-05-07

**Authors:** J. L. Hu, W. Wang, X. L. Lan, Z. C. Zeng, Y. S. Liang, Y. R. Yan, F. Y. Song, F. F. Wang, X. H. Zhu, W. J. Liao, W. T. Liao, Y. Q. Ding, L. Liang

**Affiliations:** 1 Department of Pathology, Nanfang Hospital, Southern Medical University, Guangzhou, 510515 Guangdong Province, People’s Republic of China; 2 Department of Pathology, Southern Medical University, Guangzhou, 510515 Guangdong Province, People’s Republic of China; 3 Guangdong Province Key Laboratory of Molecular Tumor Pathology, Guangzhou, 510515 Guangdong province, People’s Republic of China; 4 Department of General Surgery, Nanfang Hospital, Southern Medical University, Guangzhou, 510515 Guangdong province, People’s Republic of China; 5 Department of Oncology, Nanfang Hospital, Southern Medical University, Guangzhou, 510515 Guangdong province, People’s Republic of China

**Keywords:** Colorectal cancer, Exosomes, miR-92a-3p, Stemness, Metastasis, Chemotherapy resistance

## Abstract

**Background:**

Cancer associated fibroblasts (CAFs) are key stroma cells that play dominant roles in tumor progression. However, the CAFs-derived molecular determinants that regulate colorectal cancer (CRC) metastasis and chemoresistance have not been fully characterized.

**Methods:**

CAFs and NFs were obtained from fresh CRC and adjacent normal tissues. Exosomes were isolated from conditioned medium and serum of CRC patients using ultracentrifugation method and ExoQuick Exosome Precipitation Solution kit, and characterized by transmission electronic microscopy, nanosight and western blot. MicroRNA microarray was employed to identify differentially expressed miRNAs in exosomes secreted by CAFs or NFs. The internalization of exosomes, transfer of miR-92a-3p was observed by immunofluorescence. Boyden chamber migration and invasion, cell counting kit-8, flow cytometry, plate colony formation, sphere formation assays, tail vein injection and primary colon cancer liver metastasis assays were employed to explore the effect of NFs, CAFs and exosomes secreted by them on epithelial-mesenchymal transition, stemness, metastasis and chemotherapy resistance of CRC. Luciferase report assay, real-time qPCR, western blot, immunofluorescence, and immunohistochemistry staining were employed to explore the regulation of CRC metastasis and chemotherapy resistance by miR-92a-3p, FBXW7 and MOAP1.

**Results:**

CAFs promote the stemness, epithelial-mesenchymal transition (EMT), metastasis and chemotherapy resistance of CRC cells. Importantly, CAFs exert their roles by directly transferring exosomes to CRC cells, leading to a significant increase of miR-92a-3p level in CRC cells. Mechanically, increased expression of miR-92a-3p activates Wnt/β-catenin pathway and inhibits mitochondrial apoptosis by directly inhibiting FBXW7 and MOAP1, contributing to cell stemness, EMT, metastasis and 5-FU/L-OHP resistance in CRC. Clinically, miR-92a-3p expression is significantly increased in CRC tissues and negatively correlated with the levels of FBXW7 and MOAP1 in CRC specimens, and high expression of exosomal miR-92a-3p in serum was highly linked with metastasis and chemotherapy resistance in CRC patients.

**Conclusions:**

CAFs secreted exosomes promote metastasis and chemotherapy resistance of CRC. Inhibiting exosomal miR-92a-3p provides an alternative modality for the prediction and treatment of metastasis and chemotherapy resistance in CRC.

**Electronic supplementary material:**

The online version of this article (10.1186/s12943-019-1019-x) contains supplementary material, which is available to authorized users.

## Introduction

Colorectal cancer (CRC) is the third most common malignancy and the fourth leading cause of cancer-related deaths worldwide [1]. Due to the portal venous drainage, liver metastasis has been the most frequent form and predominant reason for CRC patients’ death. Although CRC patients with liver metastasis initially benefit from fluorouracil-and platinum-based chemotherapy, most of them experience chemotherapy resistance due to intrinsic or acquired resistance and the median survival is only about twenty months [2]. However, the mechanisms of CRC metastasis and chemotherapy resistance remain unclear.

Accumulating evidence have shown that the cellular interaction between cancer cells and surrounding stroma cells in tumor microenvironment (TME) play important roles in regulating cancer progression and therapy response [3–5]. CAFs are vital constituents of the TME that interact with cancer cells to promote tumorigenesis and progression. With the unraveling of the relationship between CAFs and tumors, CAFs are now being recognized as potential targets for anti-cancer therapy. However, the mechanisms of CAFs promoting cancer metastasis and chemotherapy resistance, as well as the communication between CAFs and cancer cells remain to be investigated.

Exosomes are microvesicles composed of lipid bilayer and contain various bioactive molecules, including DNA, microRNAs, proteins and lipids. Cells secreted exosomes can function as vital mediators between cancer cell and stroma intercellular communication by transferring genetic message associated contents in TME [6]. MicroRNAs (miRNAs) are a class of 18–22 nucleotides small single-stranded non-coding RNA molecules that promote mRNA cleavage and subsequent degradation by binding to the complementary 3′ untranslated region (UTR) of the mRNA [7]. Accumulating evidences have shown that miRNAs were involved with the regulation of cell proliferation, differentiation, metabolism and apoptosis [8]. However, the mechanisms of exosomes in regulating miRNA expression alterations and functional changes in cancer cells are still waiting for exposure.

In this study, we identify that CAFs promote the stemness, EMT, metastasis and chemoresistance of CRC cells by secreting exosomes to increase miR-92a-3p in CRC cells. Increased expression of miR-92a-3p in CRC cells activates Wnt/β-catenin pathway and inhibits mitochondrial apoptosis by directly inhibiting FBXW7 and MOAP1, contributing to cancer progression and chemotherapy resistance. Clinically, miR-92a-3p expression correlated negatively with the levels of FBXW7 and MOAP1, and high expression of exosomal miR-92a-3p in serum was closely linked with metastasis and chemotherapy resistance in CRC patients.

## Results

### CAFs secreted exosomes promote metastasis and 5-FU/L-OHP resistance of CRC cells

CAFs and NFs were obtained from colorectal cancer tissues and corresponding normal colorectal mucosa in the study (Additional file 1: Figure S1A). CAFs were positive for alpha-smooth muscle actin (α-SMA), fibroblast activation protein (FAP), fibroblast specific protein 1 (FSP-1) and mesenchymal marker vimentin, while NFs weakly expressed these proteins (Additional file 1: Figure S1B-D). To explore the roles of CAFs during CRC progression, CRC cells were treated with conditioned medium of CAFs (CAFs-CM) or NFs (NFs-CM) prior to in vitro functional experiment. Compared to human CRC SW480, SW620 and LOVO cells treated with NFs-CM, cells treated with CAFs-CM showed increased ability of migration and invasion (Additional file 1: Figure S2A). Moreover, CRC cells treated with CAFs-CM showed increased 5-FU/L-OHP therapy resistance compared to those treated with NFs-CM (Additional file 1: Figure S2B-E, **P < 0.01).

**Fig. 1:**
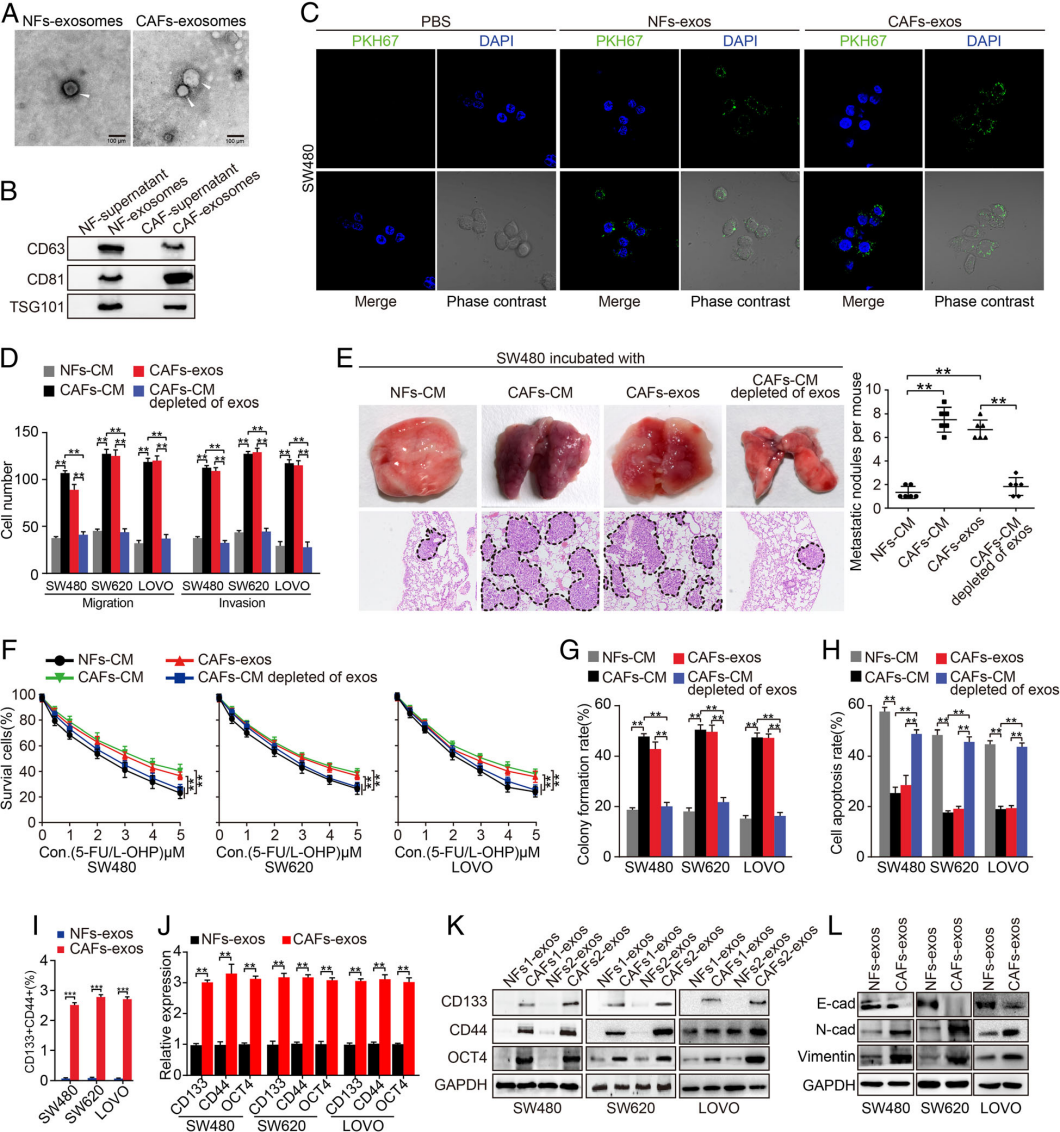
CAFs-derived exosomes promote invasion, metastasis, and chemotherapy resistance in CRC. **a** Transmission electron micrograph of NFs-derived exosomes (NFs-exos) and CAFs-derived exosomes (CAFs-exos). White arrow head points at exosomes. **b** Exosomes markers CD63, CD81 and TSG101 proteins were detected by western blot assay in NFs-exos, CAFs-exos, and the corresponding supernatant of NFs-CM and CAFs-CM obtained through ultracentrifugation. **c** Internalization of exosomes by SW480 cells examined by laser scanning confocal microscope. **d** Effect of NFs-CM, CAFs-CM, CAFs-exos, and CAFs-exos depleted of exosomes on migration and invasion of SW480, SW620 and LOVO cells by Boyden chamber assay. **e** Effect of NFs-CM, CAFs-CM, CAFs-exos, and CAFs-CM depleted of exos on the formation of metastasis nodules in lung. **f**-**h** Effect of NFs-CM, CAFs-CM, CAFs-exos, CAFs-CM depleted of exosomes on abilities of cell survival **f**, colony formation **g** and apoptosis **h** of SW480, SW620 and LOVO cells by CCK-8, colony formation and flow cytometry assays. **i** Effect of NFs-exos and CAFs-exos on percentage of CD133^+^CD44^+^ in SW480, SW620 and LOVO cells by flow cytometry assay. **j**-**l** Effect of NFs-exos and CAFs-exos on the expressions of stemness markers CD133, CD44, OCT4 **j**, **k** and EMT markers (**l** in SW480, SW620 and LOVO cells by real-time PCR and western blot assays

Recent evidence demonstrated that exosomes secreted by a variety of cells were implicated in tumor metastasis and chemotherapy resistance [9, 10]. We speculated that CAFs might exert their effects on CRC cells by secreting exosomes into the culture medium in our experiment system. To verify this conjecture, we isolated exosomes from CAFs-CM and NFs-CM using differential ultracentrifugation (Additional file 1: Figure S3A). Transmission electron microscopy (TEM) revealed cup-shaped structures (Fig. 1a), and nanosight analysis showed a mean particle size of 50–100 nm diameter structures that are typical of exosomes (Additional file 1: Figure S3B). Moreover, exosome markers CD63, CD81, and TSG101 proteins were positively expressed in these vesicles (Fig. 1b, Additional file 1: Figure S3C). After that, we labeled CAFs-secreted exosomes (CAFs-exos) or NFs-secreted exosomes (NFs-exos) with fluorescent dye PKH67 and added them into CRC culture medium to track whether these exosomes could be internalized by CRC cells. As expected, green fluorescence signals were observed in NFs-exos and CAFs-exos treated SW480, SW620 and LOVO cells by laser scanning confocal microscope (LSCM), while no fluorescence signals were observed in PBS treated cells (Fig. 1c, Additional file 1: Figure S3D), suggesting the internalization of PKH67 labeled-exosomes by CRC cells. We further explored the internalization efficiency of CAFs-exos and NFs-exos by CRC cells. CRC cells were incubated with exosomes and the percentage of cells with fluorescence signals at different time points were used to evaluate CRC cells’ internalization efficiency of CAFs-exos and NFs-exos by LSCM. The uptake efficiency of CAFs-exos and NFs-exos by CRC cells increased in a time-dependent manner and more than 90% of SW480, SW620 and LOVO cells were positive for PKH67 fluorescence at 24 h (Additional file 1: Figure S3E). No significant difference was found between the internalization of NFs-exos and CAFs-exos by CRC cells (Additional file 1: Figure S3E, both *P* > 0.05).

In vitro and in vivo experiments were performed to evaluate the effect of NFs-CM, CAFs-CM, CAFs-exos or CAFs-CM depleted of exosomes treatment on CRC cells. Boyden chamber migration and matrigel invasion assay showed that both CAFs-CM and CAFs-exos significantly increased migration and invasion abilities of CRC cells compared to NFs-CM. However, CRC cells’ migration and invasion abilities were significantly decreased when cells were treated with CAFs-CM depleted of exosomes (Fig. 1d, Additional file 1: Figure S4A, **P < 0.01). The in vivo roles of CAFs-exos were further examined in mice models. CRC cells treated with CAFs-CM and CAFs-exos formed more and larger lung metastasis nodules compared with those treated with NFs-CM. Depletion of exosomes from CAFs-CM suppressed the formation of lung metastasis nodules (Fig. 1e, **P < 0.01).

The chemotherapy sensitivity of CRC cells to clinical drugs 5-FU/L-OHP was also detected in our experimental system. CAFs-CM treated CRC cells had higher abilities of survival and colony formation and lower cell apoptosis compared to NFs-CM-treated cells under 5-FU/L-OHP therapy (Fig. 1f-h, S4B, S4C, *P < 0.05, **P < 0.01). Furthermore, we isolated exosomes from CAFs-CM and found that CAFs-exos treatment could also increase survival and colony formation abilities and decreased cell apoptosis of CRC cells compared to those of NFs-CM-treated cells under 5-FU/L-OHP therapy (Fig. 1f-h, Additional file 1: Figure S4B, C, **P < 0.01). However, depletion of exosomes in CAFs-CM could abolish the protection of CAFs-CM and CAFs-exos treatment on CRC cells under 5-FU/L-OHP therapy (Fig. 1f-h, Additional file 1: Figure S4B, C, **P < 0.01).

Accumulating evidences indicate that cancer stem cells play important roles during metastasis and chemoresistance of many human tumors. In our study, we found that CAFs-exos treated SW480, SW620 and LOVO cells (named SW480^CAFs-exos^, SW620^CAFs-exos^ and LOVO^CAFs-exos^ cells) formed larger spheres compared to cells treated with NFs-exos (named SW480^NFs-exos^, SW620^NFs-exos^ and LOVO^NFs-exos^ cells) (Additional file 1: Figure S4D, **P < 0.01). Moreover, SW480^CAFs-exos^, SW620^CAFs-exos^ and LOVO^CAFs-exos^ cells showed higher proportion of CRC^CD133+/CD44+^ cells compared to SW480^NFs-exos^, SW620^NFs-exos^ and LOVO^NFs-exos^ cells (Fig. 1i, Additional file 1: Figure S4E). Consistently, cancer stemness markers CD133, CD44, and OCT4 were obviously increased in SW480^CAFs-exos^, SW620^CAFs-exos^ and LOVO^CAFs-exos^ cells by real-time PCR, western blot and immunofluorescence assays (Fig. 1j, k, Additional file 1: Figure S4F), indicating the enhancement of CRC cell stemness phenotypes by CAFs-exos. We also found that SW480^CAFs-exos^, SW620^CAFs-exos^ and LOVO^CAFs-exos^ cells expressed higher mesenchymal markers (N-cadherin and Vimentin) and lower epithelial markers (E-cadherin) compared to SW480^NFs-exos^, SW620^NFs-exos^ and LOVO^NFs-exos^ cells, suggesting the induction of epithelial to mesenchymal transition in CRC cells by CAFs-exos (Fig. 1l). These results reveal that CAFs secreted exosomes can promote metastasis and 5-FU/L-OHP resistance, possibly by enhancing stemness and EMT in CRC.

### Direct transfer of miR-92a-3p from CAFs to CRC cells

**Fig. 2:**
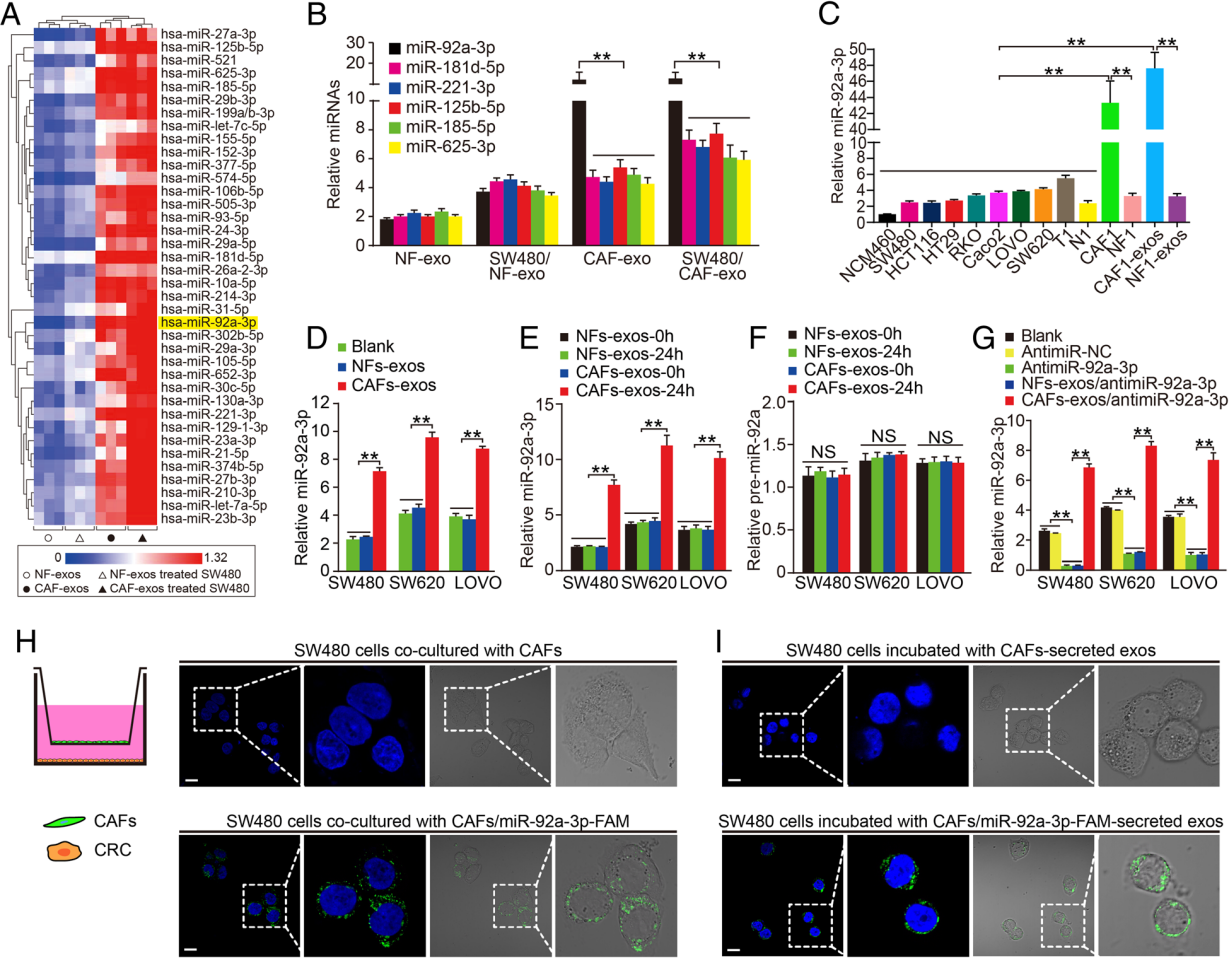
Direct transfer of CAFs secreted exosomal miR-92a-3p to CRC cells. **a** Hierarchical clustering analysis of differentially expressing miRNAs among NFs-exos, CAFs-exos, SW480^NFs-exos^ and SW480^CAFs-exos^ using microRNA microarray. **b** Relative expression of miR-92a-3p, miR-181d-5p, miR-221–3p, miR-125b-5p, miR-185-5p and miR-625-3p in NF-exos, CAFs-exos, SW480^NFs-exos^ and SW480^CAFs-exos^ cells by real-time PCR assay. U6 was used as internal control. **c** Relative expression of miR-92a-3p in normal colorectal cell NCM460, CRC cell line cells, matched CRC tissues and normal colorectal mucosa, CAFs and NFs, CAFs-exos and NFs-exos from a same CRC patient by real-time PCR analysis. U6 was used as internal control. **d** Relative expression of miR-92a-3p in Blank, SW480^CAFs-exos^, SW480^NFs-exos^, SW620^CAFs-exos^, SW620^NFs-exos^, LOVO^CAFs-exos^ and LOVO^NFs-exos^ cells by real-time PCR assay. U6 was used as internal control. **e** & **f** Relative expression of miR-92a-3p **e** and pre-miR-92a-3p **f** in SW480^CAFs-exos^, SW480^NFs-exos^, SW620^CAFs-exos^, SW620^NFs-exos^, LOVO^CAFs-exos^ and LOVO^NFs-exos^ cells at indicated time by real-time PCR assay. U6 was used as internal control. **g** Relative expression of miR-92a-3p in SW480, SW620 and LOVO cells treated with antimiR-NC, antimiR-92a-3p, antimiR-92a-3p + NFs-exos, antimiR-92a-3p + CAFs-exos by real-time PCR analysis. U6 was used as internal control. **h** Transwell co-culture of SW480 cells with CAFs and CAFs/miR-92a-3p-FAM cells. Cultured SW480 cells were harvested and observed using laser scanning confocal microscope. **i** Incubation of SW480 cells with CAFs-exos and CAFs/miR-92a-3p-FAM secreted exosomes. Cells were observed using laser scanning confocal microscope

Exosomes are bilateral structures that could shuttle proteins and microRNAs (miRNAs) into adjacent cells within the microenvironment. To investigate how CAF-exos exert their effects on CRC cells, NFs-exos, CAFs-exos, SW480^NFs-exos^, and SW480^CAFs-exos^ cells were sequenced using miRNA microarray assay (Fig. 2a). Among the differentially expressed miRNAs, the levels of miR-92a-3p, miR-181d, miR-221, miR-125b, miR-185, and miR-625 were significantly increased in CAFs-exos and SW480^CAFs-exos^ cells, which were further validated by real-time PCR (Fig. 2b). We focused on miR-92a-3p in the following experiments because its expression was highest in CAFs-exos and SW480^CAFs-exos^ cells and the underlying mechanisms of miR-92a-3p in the regulation of CRC aggressiveness and chemotherapy resistance remain to be characterized. The endogenous miR-92a-3p level was detected in normal colorectal mucosa cell line NCM460, seven human CRC cell lines, human CRC tissues and matched normal colorectal mucosa, CAFs, NFs, CAFs-exos and NFs-exos. Real-time PCR results showed that the level of miR-92a-3p was significantly higher in CAFs and CAF-exos compared to NFs, NFs-exos, CRC tissues, cell lines, and was lowest in NCM460 cells (Fig. 2c, **P < 0.01).

To determine whether CAFs-exos increase miR-92a-3p level in CRC cells, the expression of miR-92a-3p was determined in CRC cells incubated with CAFs-exos or NFs-exos. MiR-92a-3p level was significantly increased in SW480^CAFs-exos^, SW620^CAFs-exos^ and LOVO^CAFs-exos^ cells, but not in SW480^NFs-exos^, SW620^NFs-exos^ and LOVO^NFs-exos^ cells, suggesting CAFs-exos were associated with the increase of miR-92a-3p in CRC cells (Fig. 2d, **P < 0.01). To determine how miR-92a-3p was increased in CRC cells, CRC cells were incubated with CAFs-exos or NFs-exos. Real-time PCR assay showed that miR-92a-3p was significantly increased in CRC cells incubated with CAFs-exos for 24 h (Fig. 2e, **P < 0.01). Moreover, we detected the level of pre-miR-92a (precursor of miR-92a-3p) and found it was unchanged in CRC cells incubated with NFs-exos or CAFs-exos (Fig. 2f*, P* > 0.05), suggesting that the increase of miR-92a-3p in CRC cells was not the result of miRNA endogenous synthesis but more likely a direct transfer by CAFs-exos.

Further efforts were made to explore whether the increase of miR-92a-3p in CRC cells was caused by direct exosomal transfer from CAFs to CRC cells. CRC cells were firstly transfected with miR-92a-3p sponge or miR-NC prior to incubation with NFs-exos or CAFs-exos (Fig. 2g). MiR-92a-3p was significantly decreased in miR-92a-3p-sponge transfected cells. However, the level of miR-92a-3p in these cells was obviously increased after incubation with CAFs-exos rather than NFs-exos (Fig. 2g, **P < 0.01). We further co-cultured SW480 cells with control CAFs or CAFs that were transiently transfected with fluorescein amidite (FAM)-tagged miR-92a-3p for 24 h using a transwell co-culture system (Fig. 2h). Interestingly, green fluorescence signals of miR-92a-3p-FAM were observed in SW480 cells co-cultured with miR-92a-3p-FAM expressing CAFs, rather than in SW480 cells co-cultured with control CAFs (Fig. 2h). To further determine whether the transfer of miR-92a-3p is mediated by exosomes, SW480 cells were incubated with exosomes derived from the conditioned medium of miR-92a-3p-FAM expressing CAFs or control CAFs. Consistently, fluorescently labelled green signals were also observed in SW480 cells incubated with exosomes secreted by miR-92a-3p-FAM expressing CAFs, while no fluorescent signals were observed in SW480 cells incubated with exosomes secreted by control CAFs (Fig. 2i), confirming that miR-92a-3p was directly transferred from CAFs to CRC cells via exosomes.

### CAFs-secreted miR-92a-3p enhances stemness, EMT, metastasis and 5-FU/L-OHP resistance of CRC

**Fig. 3:**
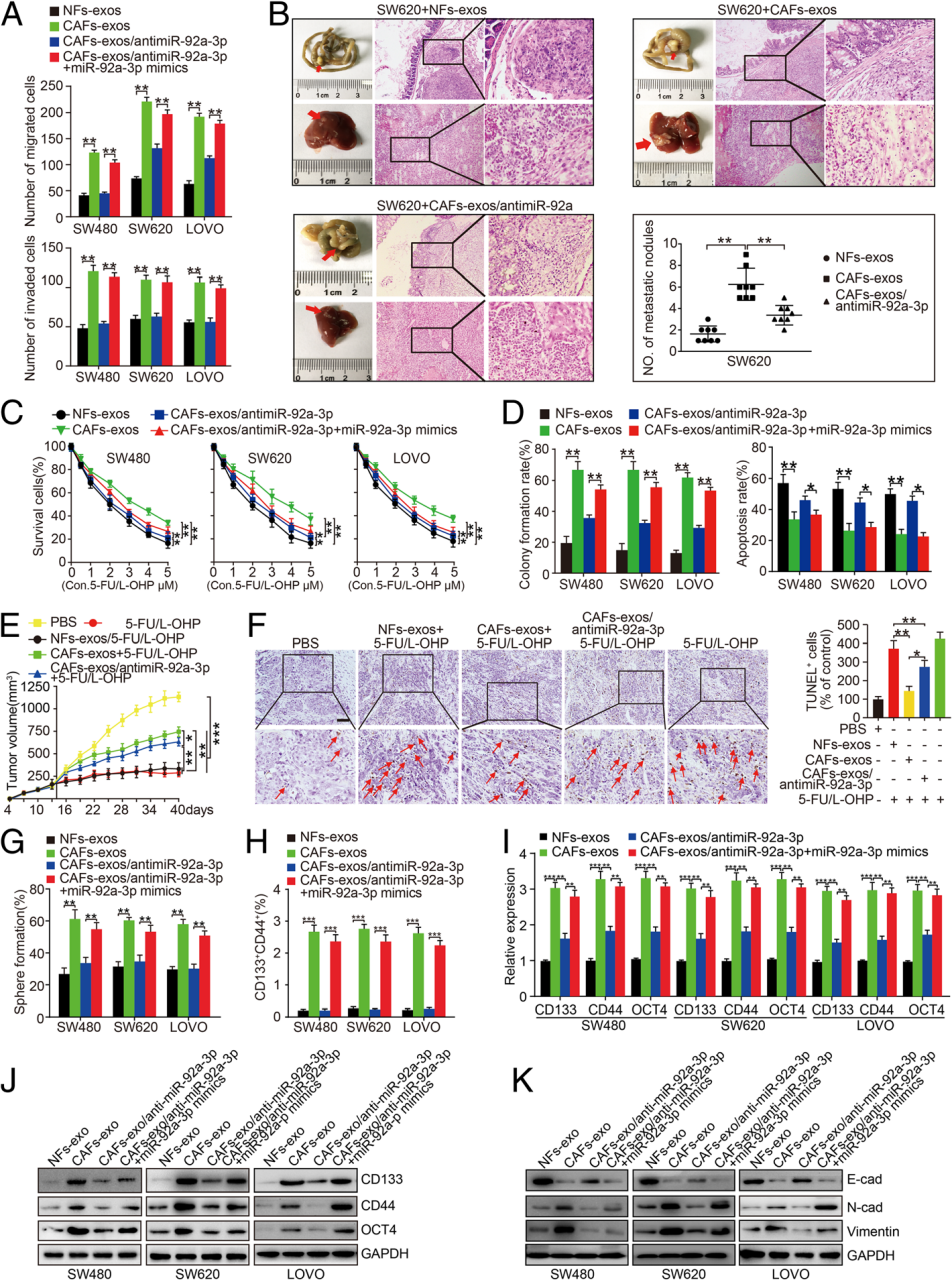
CAFs secreted exosomal miR-92a-3p promoted metastasis and chemotherapy resistance of CRC. **a** Effects of NFs-exos, CAFs-exos, CAFs-exos/antimiR-92a-3p, CAFs-exos/antimiR-92a-3p + miR-92a-3p mimics treatment on cell migration and invasion of SW480, SW620 and LOVO cells. **b** Gross and microscopy observations of primary colon tumors and liver metastases in mice injected with SW620^NFs-exos^, SW620^CAFs-exos^, SW620^CAFs-exos^/antimiR-92a-3p *n* = 8). The number of metastatic nodules in individual mice was counted under the microscope. The liver sections were stained with H&E. (C&D) Effects of NFs-exos, CAFs-exos, CAFs-exos/antimiR-92a-3p, CAFs-exos/antimiR-92a-3p + miR-92a-3p mimics treatment on abilities of cell survival **c**, colony formation and apoptosis **d** of SW480, SW620 and LOVO cells. **e** SW480 cells were injected into the flank of mice to establish xenografts. 5-FU/L-OHP (5 mg/kg) or same volume of PBS every 3 days were injected subsequently. When tumors formed, NFs-exos, CAFs-exos, and CAFs-exos/antimiR-92a-3p were injected into the vicinity of the subcutaneous tumors every 3 days. Tumor volume was calculated using the formula V = length × width^2^/2. **f** Effect of PBS, 5-FU/L-OHP, NFs-exos/5-FU/L-OHP, CAFs-exos/5-FU/L-OHP, and CAFs-exos/antimiR-92a-3p + 5-FU/L-OHP treatment on SW480 cells derived tumor apoptosis assessed by TUNEL assay. Red arrows pointed out the apoptotic cells in indicated group of tumors. **g**-**k** Effect of NFs-exos, CAFs-exos, CAFs-exos/antimiR-92a-3p and CAFs-exos/antimiR-92a-3p + miR-92a-3p mimics treatment on sphere formation ability **g**, percentage of CD133^+^CD44^+^ cells **h**, CD133, CD44, OCT4 **i** & **j** and EMT markers expression **k** in SW480, SW620 and LOVO cells using sphere formation, flow cytometry, real-time PCR and western blot assays. GAPDH was used as internal control

We then set out to explore the role of CAFs-exosomal miR-92a-3p in the aggressiveness of CRC cells. Boyden chamber assay showed that the number of migrated and invaded SW480^CAFs-exos^, SW620^CAFs-exos^ and LOVO^CAFs-exos^ cells was increased compared to SW480^NFs-exos^, SW620^NFs-exos^ and LOVO^NFs-exos^ cells, while transfection of miR-92a-3p-sponge into CAFs-exos could significantly reduce the migration and invasion of SW480^CAFs-exos^, SW620^CAFs-exos^ and LOVO^CAFs-exos^ cells. However, reintroduction of miR-92a-3p mimics into CRC cells could reverse miR-92a-3p-sponge mediated inhibition of cell migration and invasion (Fig. 3a, Additional file 1: Figure S5A, B, ** *P* < 0.01), suggesting CAFs-secreted exosomal miR-92a-3p could significantly enhance cell migration and invasion of CRC.

To further explore the effect of CAFs-secreted miR-92a-3p in vivo, SW620 cells were firstly injected in the flank of mice to form subcutaneous tumors. Tumors were then resected, minced, and transplanted to the mucosa of ileocecal junction. Macroscopy results showed that tumors were formed in the ileocecal junction and liver. Moreover, the number of liver metastases in mice injected with SW620^CAFs-exos^ cells was more than those injected with SW620^NFs-exos^ cells, while injection of SW620 cells treated with CAFs-exos transfected with miR-92a-3p-sponge could reduce the number of liver metastases compared to control (Fig. 3b, **P < 0.01).

The effect of CAFs-exosomal miR-92a-3p on CRC cells response to 5-FU/L-OHP therapy was also determined in vitro and in vivo. SW480^CAFs-exos^, SW620^CAFs-exos^ and LOVO^CAFs-exos^ cells showed higher survival and colony formation abilities and lower cell apoptosis compared to cells treated with NFs-exos. Suppressing miR-92a-3p in CAFs-exos could significantly inhibit survival and colony formation and induce cell apoptosis of SW480^CAFs-exos^, SW620^CAFs-exos^ and LOVO^CAFs-exos^ cells. Re-introduction of miR-92a-3p mimics into CRC cells reversed these effects (Fig. 3c, d, Additional file 1: Figure S5C, D, *P < 0.05, **P < 0.01). In vivo experiment showed that the size of subcutaneous tumors derived from SW480 cells treated with PBS was the largest, while nude mice injected with SW480 cells under 5-FU/L-OHP therapy formed the smallest tumors. Importantly, tumors derived from SW480^CAFs-exos-miR-92a-3p-sponge^ cells were smaller than CAFs-exos groups and larger than NFs-exos group under 5-FU/L-OHP therapy (Fig. 3e, Additional file 1: Figure S5E, **P < 0.01,). Moreover, tumors derived from SW480^CAFs-exos^ cells yielded decreased apoptosis compared to those of SW480^NFs-exos^ group. Inhibition of miR-92a-3p in CAFs-exos could induce cell apoptosis (Fig. 3f, *P < 0.05, ** *P* < 0.01).

In addition, SW480^CAFs-exos^, SW620^CAFs-exos^ and LOVO^CAFs-exos^ cells formed more and larger spheres compared to those formed by SW480^NFs-exos^, SW620^NFs-exos^ and LOVO^NFs-exos^ cells. Transfection of CAFs-exos with miR-92a-3p-sponge significantly reduced the number and size of spheres, while re-introduction of miR-92a-3p mimics in CRC cells could increase spheres formation (Fig. 3g, Additional file 1: Figure S6A, **P < 0.01). Consistently, SW480^CAFs-exos^, SW620^CAFs-exos^ and LOVO^CAFs-exos^ cells showed higher proportion of CRC^CD133+/CD44+^ cells compared to SW480^NFs-exos^, SW620^NFs-exos^ and LOVO^NFs-exos^ cells. Suppressing miR-92a-3p in CAFs-exos decreased the proportion of CRC^CD133+/CD44+^ cells while reintroduction of miR-92a-3p increased CRC^CD133+/CD44+^ cell proportion (Fig. 3h, Additional file 1: Figure S6B, *** *P* < 0.001). Moreover, cell stemness markers CD133, CD44 and OCT4 were obviously increased in SW480^CAFs-exos^, SW620^CAFs-exos^ and LOVO^CAFs-exos^ cells (Fig. 3i, j, S6C, **P < 0.01, ***P < 0.001). Transfection of CAFs-exos with miR-92a-3p-sponge could decrease CD133, CD44 and OCT4, while re-introduction of miR-92a-3p mimics in CRC cells could increase these proteins in CRC cells (Fig. 3i, j, Additional file 1: Figure S6C). Moreover, SW480^CAFs-exos^, SW620^CAFs-exos^ and LOVO^CAFs-exos^ cells expressed lower epithelial marker E-cadherin but higher mesenchymal markers N-cadherin and vimentin compared to SW480^NFs-exos^, SW620^NFs-exos^ and LOVO^NFs-exos^ cells. However, suppressing miR-92a-3p in CAFs-exos increased E-cadherin and decreased N-cadherin and vimentin in CRC cells. Reintroduction of miR-92a-3p in CRC cells could largely enhance the N-cadherin and vimentin and inhibit E-cadherin expression in CRC cells (Fig. 3k). These above data demonstrate that CAFs promote metastasis and 5-FU/L-OHP resistance and enhance stemness and EMT in CRC by transferring exosomal miR-92a-3p to CRC cells.

### FBXW7 and MOAP1 are downstream targets of miR-92a-3p

**Fig. 4:**
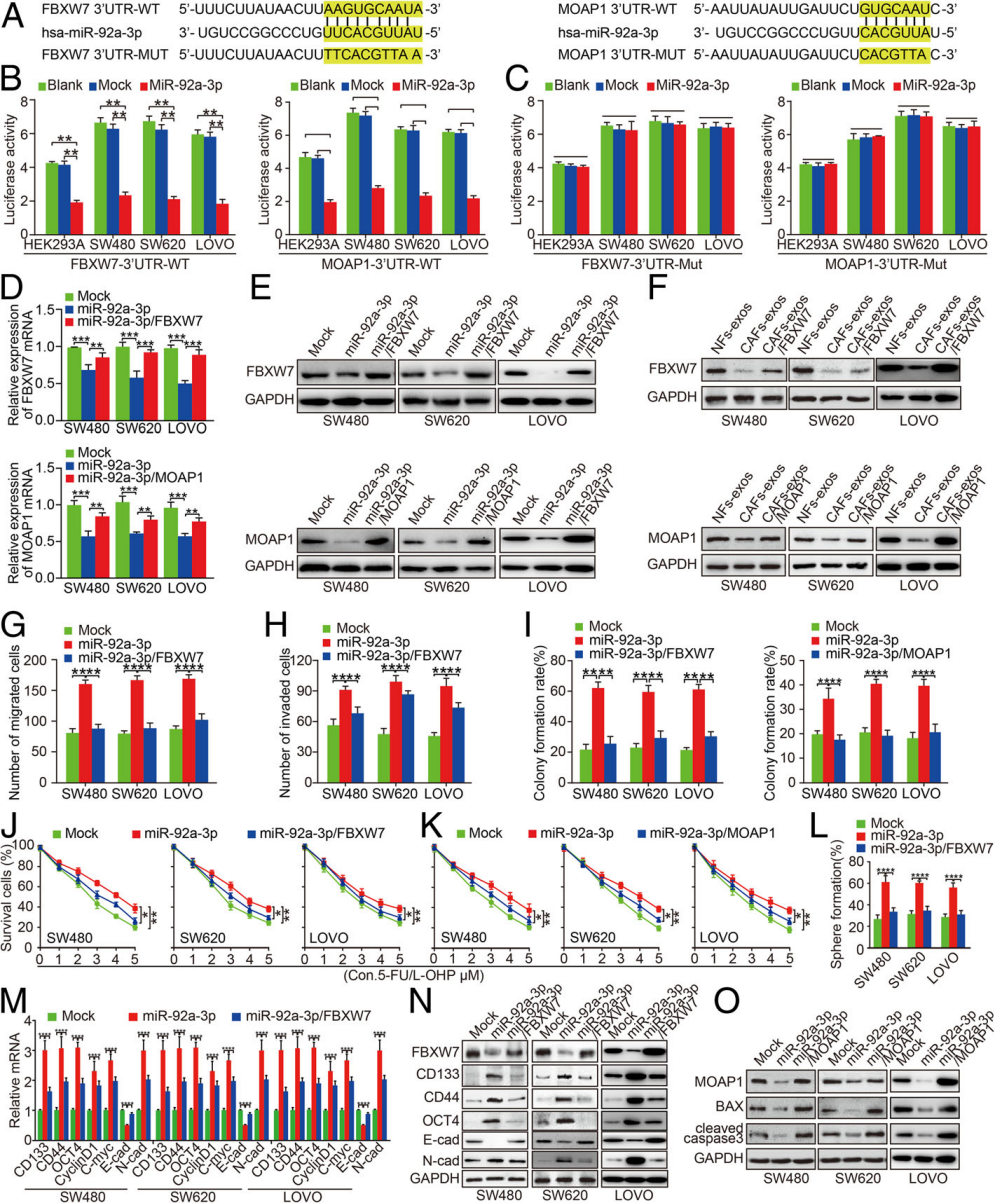
FBXW7 and MOAP1 attenuate miR-92a-3p-mediated promotion of aggressiveness and chemotherapy resistance of CRC. **a** Sequences of miR-92a-3p and the potential miR-92a-3p-binding sites at the 3’UTR of FBXW7 and MOAP1. Also shown are nucleotides mutated in FBXW7–3′-UTR mutant and MOAP1–3′-UTR mutant. Seed sequences are marked. **b** & **c** Effect of Blank, Mock and ectopic miR-92a-3p expression on the luciferase activity of FBXW7 3’UTR wild type **b**, FBXW7 3’UTR mutation **c**, MOAP1 3’UTR wild type **b**, and MOAP1 3’UTR mutation **c** in HEK293A, SW480, SW620 and LOVO cells by dual-luciferase reported assay. **d**&**e** Expression of FBXW7 and MOAP1 in SW480, SW620 and LOVO cells transfected with Mock, miR-92a-3p, miR-92a-3p/FBXW7, and miR-92a-3p/MOAP1 by real-time PCR **d** and western blot **e** assays. GAPDH was used as internal control. **f** Expression of FBXW7 and MOAP1 in SW480, SW620 and LOVO cells treated with NFs-exos, CAFs-exos, CAFs-exos/FBXW7, CAFs-exos/MOAP1 by western blot. GAPDH was used as internal control. **g**&**h** Effect of Mock, miR-92a-3p, miR-92a-3p/FBXW7 treatment on migration **g** and invasion **h** of SW480, SW620 and LOVO cells by Boyden chamber. **i** Effect of Mock, miR-92a-3p, miR-92a-3p/FBXW7, and miR-92a-3p/MOAP1 treatment on colony formation ability of SW480, SW620 and LOVO cells by plate colony formation assay. **j** & **k** Effect of Mock, miR-92a-3p, miR-92a-3p/FBXW7 **j**, miR-92a-3p/MOAP1 **k** treatment on survival of SW480, SW620 and LOVO cells by CCK-8 assay. **l** Effect of Mock, miR-92a-3p, miR-92a-3p/FBXW7 treatment on sphere formation of SW480, SW620 and LOVO cells by spheres formation assay. **m** Effect of Mock, miR-92a-3p, miR-92a-3p/FBXW7 transfection on the expression of CD133, CD44, OCT4, CyclinD1, C-myc, E-cad and N-cad expression in SW480, SW620 and LOVO cells by real-time PCR. GAPDH was used as internal control. **n** Effect of Mock, miR-92a-3p, miR-92a-3p/FBXW7 treatment on the expression of FBXW7, CD133, CD44, OCT4, E-cad and N-cad expression in SW480, SW620 and LOVO cells by western blot. GAPDH was used as internal control. **o** Effect of Mock, miR-92a-3p, miR-92a-3p/MOAP1 transfection on the expression of MOAP1, BAX and cleaved caspase3 in SW480, SW620 and LOVO cells by western blot. GAPDH was used as internal control

To screen the downstream targets of miR-92a-3p, we utilized five bioinformatics and online prediction database (DIANAmT, miRanda, miRDB, miRWalk, Targetscan) and screened FBXW7 and MOAP1 as potential downstream targets of miR-92a-3p with highest predictive values. The TargetScan algorithm showed the potential complementarity sequence in the 3’UTR of FBXW7 and MOAP1 to the seed sequence of miR-92a-3p (Fig. 4a). To substantiate the site-specific repression of miR-92a-3p on FBXW7 and MOAP1, we constructed mutated FBXW7 3′-UTR and MOAP1 3′-UTR luciferase reporter. Dual-luciferase activity assay showed that the luciferase activity of FBXW7 or MOAP1 with wild type 3’UTR was significantly suppressed in miR-92a-3p expressing HEK293A, SW480, SW620 and LOVO cells (Fig. 4b, **P < 0.01). In contrast, the luciferase activity of FBXW7 or MOAP1 with 3’UTR mutation was not changed (Fig. 4c, *P* > 0.05). Moreover, FBXW7 and MOAP1 were significantly decreased in miR-92a-3p expressing CRC cells compared to Mock cells (Fig. 4d, e, ** *P* < 0.01). In addition, both FBXW7 and MOAP1 proteins were suppressed in SW480^CAFs-exos^, SW620^CAFs-exos^ and LOVO^CAFs-exos^ cells compared to SW480^NFs-exos^, SW620^NFs-exos^ and LOVO^NFs-exos^ cells while re-introduction of FBXW7 and MOAP1 in CRC cells could increase their levels (Fig. 4f). These results indicate that FBXW7 and MOAP1 are downstream targets of miR-92a-3p in CRC cells.

### FBXW7 and MOAP1 attenuate miR-92a-3p mediated stemness, metastasis and 5-FU/L-OHP resistance of CRC in vitro and in vivo

Having validated FBXW7 and MOAP1 as downstream targets of miR-92a-3p, we further explored their roles in miR-92a-3p-mediated regulation of CRC. SW480, SW620 and LOVO cells were transfected with FBXW7 and MOAP1 plasmids separately. Boyden chamber assay showed that re-introduction of FBXW7 could inhibit miR-92a-3p mediated promotion of migration and invasion in CRC cells (Fig. 4g, h, Additional file 1: Figure S7A, B, ** *P* < 0.01). In addition, re-expression of FBXW7 and MOAP1 significantly reduced colonies formation and survival abilities of CRC cells under 5-FU/L-OHP treatment (Fig. 4i, k, Additional file 1: Figure S7C, D, * *P* < 0.05, ** *P* < 0.01). Overexpression of FBXW7 inhibited sphere formation, CD133, CD44, OCT4, cyclinD1, c-myc, N-cadherin and increased E-cadherin in miR-92a-3p expressing CRC cells, leading to inhibition of cell stemness, proliferation and EMT phenotypes (Fig. 4l-n, Additional file 1: Figure S7E, F, ** *P* < 0.01). Re-introduction of MOAP1 induced the expression of apoptotic proteins BAX and cleaved caspase 3 in miR-92a-3p expressing CRC cells (Fig. 4o), thus promoting cancer cell apoptosis.

**Fig. 5:**
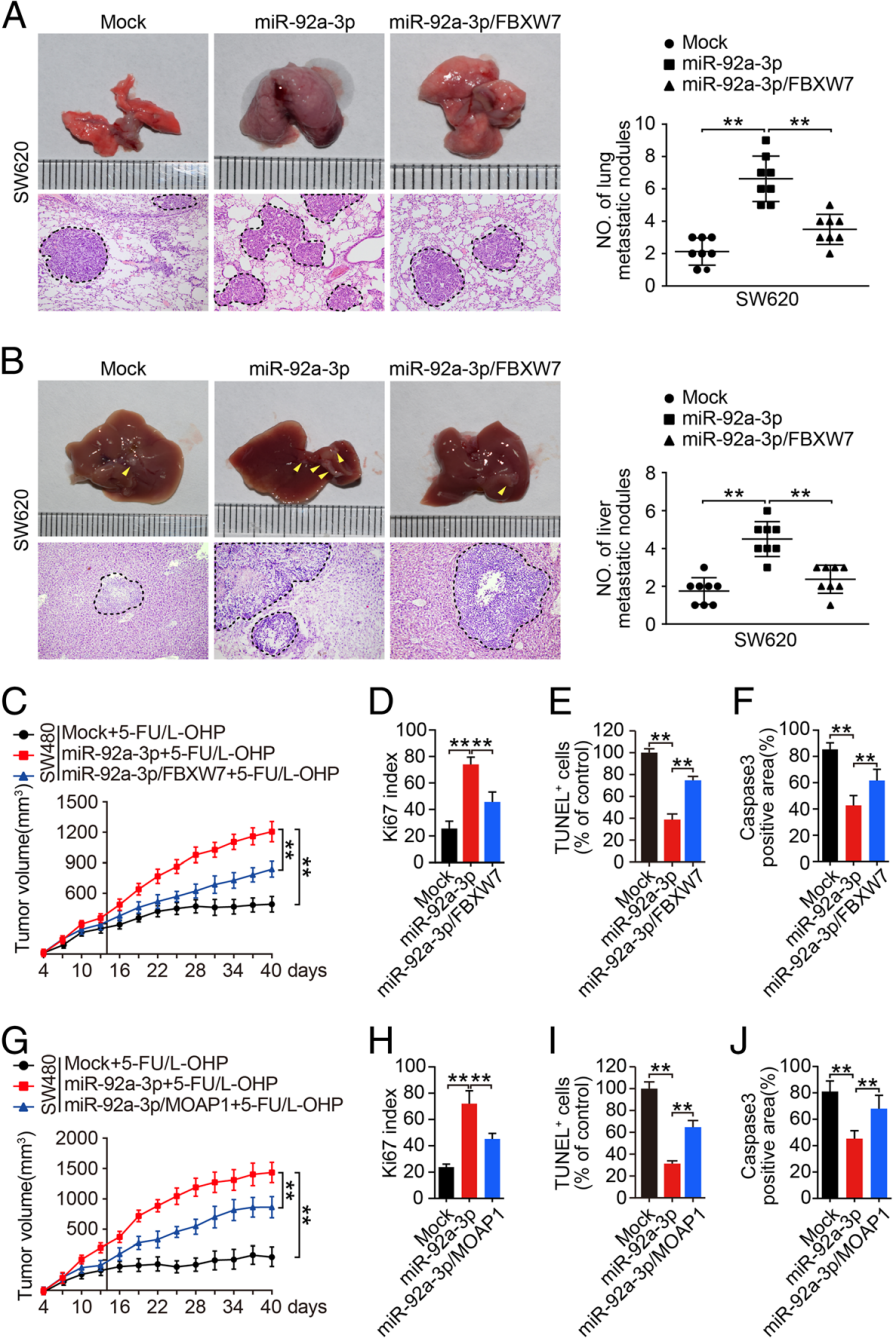
FBXW7 and MOAP1 attenuate miR-92a-3p-mediated promotion of aggressiveness and chemotherapy resistance of CRC in vivo. **a** The formation of metastatic nodules in lung derived from SW620/Mock, SW620/miR-92a-3p, and SW620/miR-92a-3p/FBXW7 cells by tail vein injection method (n = 8 in each group). **b** The formation of metastatic nodules in liver derived from SW620/Mock, SW620/miR-92a-3p, and SW620/miR-92a-3p/FBXW7 cells by subcapsular injection of the spleen method (n = 8 in each group). **c** The formation of subcutaneous tumors derived from SW480/Mock, SW480/miR-92a-3p, and SW480/miR-92a-3p/FBXW7 cells under 5-FU/L-OHP therapy by subcutaneous injection into the flank of mice *n* = 6 in each group). **d**-**f** Detection of proliferation and apoptosis by Ki-67 **d**, TUNEL **e**, and caspase3 **f** expression in tumor tissues derived from SW480/Mock, SW480/miR-92a-3p, and SW480/miR-92a-3p/FBXW7 cells by IHC and TUNEL assays. **g** The formation of subcutaneous tumors derived from SW480/Mock, SW480/miR-92a-3p, and SW480/miR-92a-3p/MOAP1 cells under 5-FU/L-OHP therapy by subcutaneous injection into the flank of mice (n = 6 in each group). **h**-**j** Detection of proliferation and apoptosis by Ki-67 (H), TUNEL **i**, and caspase3 **j** expression in tumor tissues derived from SW480/Mock, SW480/miR-92a-3p, and SW480/miR-92a-3p/MOAP1 cells by IHC and TUNEL assays

To further explore the effect of FBXW7 and MOAP1 on metastasis and 5-FU/L-OHP resistance of CRC, we constructed SW620/Mock, SW620/miR-92a-3p, SW620/miR-92a-3p/FBXW7, SW620/miR-92a-3p/MOAP1 cells by lentivirus infection. Tail vein injection and subcapsular injection of the spleen assay showed that metastatic nodules formed in lung and liver by SW620/miR-92a-3p cells were significantly increased compared to those formed by SW620/Mock cells, while the formation of metastatic nodule by SW620/miR-92a-3p/FBXW7 cells was obviously decreased compared to SW620/miR-92a-3p cells (Fig. 5a, b, ** *P* < 0.01).

Moreover, the size of subcutaneous tumors formed by SW480/miR-92a-3p was significantly larger than that formed by SW480/Mock cells (Fig. 5c, Additional file 1: Figure S8A, B, ** *P* < 0.01) under 5-FU/L-OHP therapy. However, re-introduction of FBXW7 into SW480/miR-92a-3p cells could significantly decrease SW480/miR-92a-3p cells derived subcutaneous tumors (Fig. 5c, Additional file 1: Figure S8A, ** *P* < 0.01). In addition, SW480/miR-92a-3p/FBXW7 cells derived tumors showed lower ki67 proliferation index, increased TUNEL positive apoptotic cells, and higher level of caspase3 apoptotic proteins by IHC and TUNEL assay (Fig. 5d-f, Additional file 1: Figure S8C, E, G, ** *P* < 0.01). Furthermore, we found consistent in vivo results in tumors formed by SW480/Mock, SW480/miR-92a-3p, and SW480/miR-92a-3p/MOAP1 expressing cells (Fig. 5g-j, Additional file 1: Figure S8B, D, F, H, ** *P* < 0.01). Thus, these results showed that FBXW7 and MOAP1 could attenuate miR-92a-3p mediated metastasis and chemotherapy resistance of CRC cells in vivo.

### FBXW7 and MOAP1 reverse the oncogenic role of miR-92a-3p by promoting ubiquitination degradation of β-catenin and mitochondrial apoptosis

**Fig. 6:**
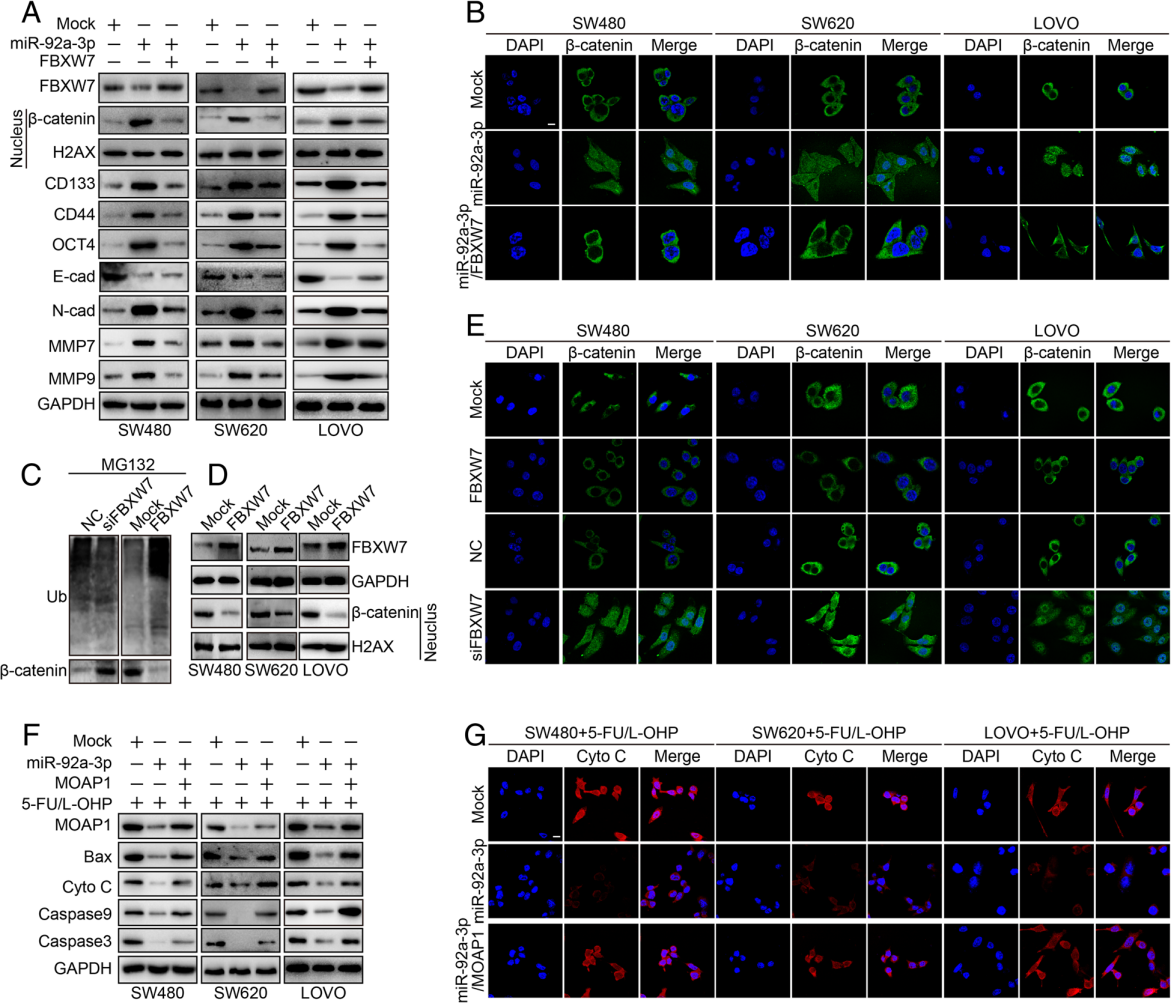
FBXW7 and MOAP1 attenuated miR-92a-3p mediated Wnt/β-catenin activation and mitochondrial inhibition in CRC. **a** Effect of Mock, miR-92a-3p, miR-92a-3p/FBXW7 treatment on Wnt/β-catenin activation, stemness and EMT markers expression in SW480, SW620 and LOVO cells by western blot assay. **b** Effect of Mock, miR-92a-3p, miR-92a-3p/FBXW7 transfection on Wnt/β-catenin activation in SW480, SW620 and LOVO cells by laser scanning confocal microscope. **c** Effect of Mock, FBXW7, NC and siFBXW7 transfection on ubiquitination and degradation of β-catenin in SW480, SW620 and LOVO cells by western blot. **d** Effect of Mock and FBXW7 transfection on expression of nucleus β-catenin in SW480, SW620 and LOVO cells by western blot assay. **e** Effect of Mock, FBXW7, NC, and siFBXW7 treatment on expression of β-catenin in SW480, SW620 and LOVO cells by laser scanning confocal microscope. **f** Effect of Mock, miR-92a-3p, MOAP1 on expression of MOAP1, BAX, cytochrome C, caspase9, caspase3 in in SW480, SW620 and LOVO cells CRC cells treated with 5-FU/L-OHP therapy. **g** Effect of Mock, miR-92a-3p, MOAP1 on expression of cytochrome C in in SW480, SW620 and LOVO cells cells treated with 5-FU/L-OHP therapy

To determine the potential mechanisms underlying the role of FBXW7 in abrogating the tumor-promoting effects of miR-92a-3p, we investigated the Wnt/β-catenin pathway. The introduction of exogenous miR-92a-3p significantly increased β-catenin expression in the nuclear fractions in SW480, SW620 and LOVO cells, whereas re-introduction of FBXW7 in miR-92a-3p expressing cells decreased the expression of β-catenin in the nuclear fractions (Fig. 6a, b). Moreover, overexpression of miR-92a-3p increased CD133, CD44, OCT4, N-cadherin, MMP7, MMP9 and decreased E-cadherin in CRC cells. In contrast, overexpression of FBXW7 in miR-92a-3p expressing cells showed the opposite effect (Fig. 6a). Furthermore, we found that FBXW7 inhibited β-catenin in the nucleus fractions by promoting β-catenin ubiquitination and degradation in CRC cells (Fig. 6b-e). We also investigated the mechanism of MOAP1 in attenuating the effects of miR-92a-3p on CRC chemotherapy resistance. Overexpression of miR-92a-3p suppressed MOAP1 as well as Bax, cytochrome c, caspase9, caspase3 in CRC cells under 5-FU/L-OHP therapy (Fig. 6f). Re-expression of MOAP1 in miR-92a-3p expressing cells showed less expression of apoptotic proteins and cytochrome c release to the cytoplasm (Fig. 6f, g).

### MiR-92a-3p negatively correlates with FBXW7 and MOAP1, and high expression of exosomal miR-92a-3p in serum predicts metastasis and chemotherapy resistance in CRC patients

**Fig. 7:**
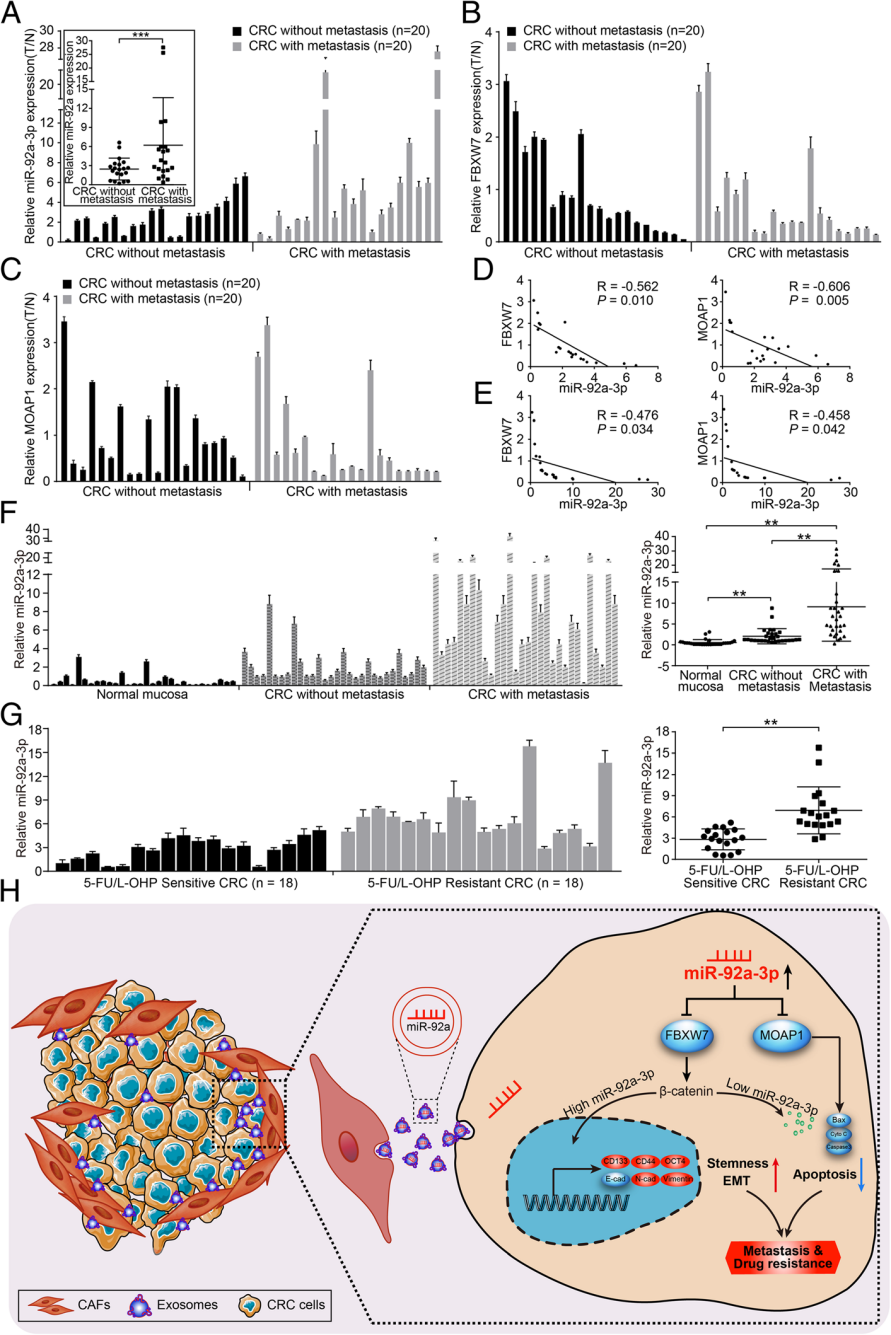
The expression of miR-92a-3p correlates negatively with FBXW7 and MOAP1 and is associated with metastasis in serum exosomes. **a** Real-time PCR analysis of miR-92a-3p, FBXW7 and MOAP1 in non-metastatic and metastatic fresh CRC tissues *n* = 20 for each group) (tumor ration to normal). The expression of miR-92a-3p was compared between CRC with metastasis and CRC without metastasis. U6 was used as internal control. **b** & **c** Real-time PCR analysis of FBXW7 **b** and MOAP1 **c** in non-metastatic and metastatic fresh CRC tissues (n = 20 for each group) (tumor ration to normal). GAPDH was used as internal control. (D&E) Pearson correlation analysis between miR-92a-3p and FBXW7 levels, miR-92a-3p and MOAP1 levels in 20 cases of non-metastatic CRC **d**, 20 cases of metastatic CRC **e** and corresponding normal colorectal mucosa. **f** Real-time PCR analysis of miR-92a-3p in serum-derived exosomes collected from 30 normal persons, 30 non-metastatic CRC patients and 30 metastatic CRC patients. U6 was used as internal control. **g** Real-time PCR analysis of miR-92a-3p in serum-derived exosomes collected from 18 cases of 5-FU/L-OHP sensitive CRC patients and 18 cases of 5-FU/L-OHP resistant CRC patients. U6 was used as internal control. **h** Briefly, Colorectal cancer (CRC) cells uptake cancer associated fibroblasts secreted exosomes, leading to an increase of miR-92a-3p and stemness, EMT, metastasis, and 5-FU/L-OHP resistance in CRC cells. Mechanically, miR-92a-3p promotes aggressiveness and chemotherapy resistance by directly binding to 3’UTR of FBXW7 and MOAP1 and suppressing their expressions in CRC cells. Re-expression of FBXW7 and MOAP1 attenuate the role of miR-92a-3p by inhibiting Wnt/β-catenin and mitochondrial apoptosis in CRC

To investigate the clinical values of miR-92a-3p for CRC, we detected its expression in 40 cases of CRC tissues and corresponding normal mucosa using real-time PCR. The expression of miR-92a-3p was higher in CRC tissues compared to normal mucosa. Moreover, miR-92a-3p was higher in CRC with metastasis compared to CRC without metastasis (Fig. 7a, *** *P* < 0.001). The expressions of FBXW7 and MOAP1 were lower in CRC tissues than in normal mucosa (Fig. 7b, c). Correlation analysis showed that miR-92a-3p was negatively correlated with FBXW7 and MOAP1 in CRC with (Fig. 7d) or without metastasis (Fig. 7e). The above results further validate that FBXW7 and MOAP1 are both downstream target genes of miR-92a-3p.

To ascertain whether exosomal miR-92a-3p is associated with CRC metastasis, we detected the level of exosomal miR-92a-3p in the serum in healthy persons, CRC patients with metastasis and those without metastasis. Results showed that exosomal miR-92a-3p level was highest in CRC patients with metastasis, gradually decreased in CRC patients without metastasis, and lowest in the healthy people (Fig. 7f). Moreover, we detected the level of exosomal miR-92a-3p in the serum of 18 cases of 5-FU/L-OHP sensitive and resistant CRC patients. Results showed that exosomal miR-92a-3p was significantly higher in 5-FU/L-OHP resistant CRC patients compared to that in 5-FU/L-OHP sensitive CRC patients (Fig. 7g). These results strongly suggest that exosomal miR-92a-3p may be a predictor of CRC metastasis and chemotherapy resistance.

## Discussion

Metastatic outgrowths are the predominant reasons for the death of human cancers, including CRC [11]. Traditionally, fluoropyimidine- and platinum-based chemotherapy is considered the first line treatment for metastatic CRC. Acquisition of resistance to multiple chemotherapies causes the therapy failure in CRC. Tumor cells and surrounding stroma, such as CAFs, macrophages, and immune cells, mutually communicate with each other and cause tumor progression and therapy resistance [12, 13]. However, the mechanisms of tumor metastasis and chemotherapy resistance remain to be explored.

In the present study, we showed that CAFs are key determinants that contribute to growth, invasion, metastasis, and therapy resistance of human colorectal cancer by exosome mediated cellular communication. Exosomes can be secreted by various cells and modulate angiogenesis, invasion and metastasis [14]. Cancer cells secreted exosomes could promote vascular permeability, pre-metastatic niche formation and chemotherapy resistance in a wide range of human tumors [15–17]. In contrast, stroma cells also enhance malignant phenotype of cancer cells by delivering tumor promoting exosomes [18]. Malignant activated platelets secreted exosomes promoted the angiogenesis, invasion and metastasis of lung cancer cells [19]. CAFs secreted exosomes enhanced the proliferation of pancreatic cells and induced gemcitabine resistance by increasing the expression of Snail [20]. CD81 positive exosomes derived from CAFs activated Wnt-planar signal pathway and promoted the migration and lung metastasis of breast cancer cells [21]. However, the effects of exosomes on cancer cell aggressiveness remain uncharacterized. Here, we showed that CAFs secreted exosomes promoted cell invasion and chemotherapy resistance by promoting cell stemness and EMT in CRC. Cancer stem cells (CSCs) are self-renewable cell types that contribute to initiation, metastasis, relapse, and chemotherapy resistance of cancer cells [22, 23]. CAFs play essential roles in promoting both differentiation of CSCs and dedifferentiation of non-CSCs toward attaining a CSC-like phenotype [24, 25]. CAFs promoted cell stemness markers CD133 and CD44 levels, increased the proportion of CD133 and CD44 positive CSC cells and induced EMT phenotypes in CRC cells, causing enhanced metastasis and chemotherapy resistance in CRC cells.

Increasing evidence demonstrate that miRNAs, small non-coding RNAs, are loaded in exosomes and can be functionally delivered to recipient cells to exert post transcriptional regulation of gene expression by binding to the complementary sequences in the 3′ untranslated regions of mRNAs [26–28]. Breast cancer secreted exosomal miR-105 destroyed endothelial barriers via targeting tight junction protein ZO-1 and promoted metastasis [15]. Exosomes derived from tamoxifen resistant breast cancer cells could elevate miR-221/222 levels and induce tamoxifen resistance in recipient ER-positive breast cancer cells [29]. MiR-92a-3p played important roles in the regulation of organ development, angiogenesis, immunity, and cancer, such as liposarcoma, breast, and gastric cancer [30–34]. Increased miR-92a-3p is associated with lymph node metastasis and worse prognosis of CRC patients [35]. However, it is still unknown why miR-92a-3p highly expressed in CRC cells. Here, we found the level of miR-92a-3p was highly expressed in CAFs and CAFs-exos. Importantly, CAFs transferred exosomes to CRC cells, causing the increase of miR-92a-3p in CRC. Moreover, CAFs secreted exosomal miR-92a-3p promoted cell stemness and EMT and inhibited cell apoptosis, leading to metastasis and chemotherapy resistance in CRC.

Furthermore, FBXW7 and MOAP1 were validated as downstream targets of miR-92a-3p in CRC. FBXW7, also known as Hcdc4, has been implicated in different human tumors. Decreased expression of FBXW7 attenuates miR-223 mediated promotion of esophagus cancer cell migration and invasion and was associated with worse outcome [36]. FBXW7 and PTEN works together to inhibit breast cancer progression by suppressing mTOR [37]. MOAP1, an important regulator of cell apoptosis, combines to BAX and induces cell apoptosis. MiR-25 promotes cell proliferation and inhibits cell apoptosis by directly targeting MOAP1 in non-small cell lung cancer [38]. Here we found that overexpression of FBXW7 and MOAP1 inhibited Wnt/β-catenin pathway activation and promoted mitochondrial apoptosis, leading to inhibition of cell stemness and promotion of cell apoptosis in CRC, thus reversed CAFs-exos mediated cell migration, invasion and chemotherapy resistance in CRC cells.

Finally, we investigated the clinical values of miR-92a-3p in the progression of CRC. MiR-92a-3p was highly expressed in the plasma and could be used as early biomarker of patients with hepatocellular carcinoma [39]. MiR-19a-3p, miR-92a-3p, miR-223-3p, and miR-422a were highly expressed in serum of CRC patients. Exosomal miR-17-92a cluster expression in serum was correlated with the recurrence of CRC. CRC patients’ serum derived exosomal miR-19a was significantly increased compared to healthy individuals. High exosomal miR-19a expression was associated with poorer prognosis of CRC patients [40]. In our study, we found that miR-92a-3p was significantly increased in CRC tissue and associated with metastasis of CRC patients. Moreover, we found that exosomal miR-92a-3p was highly expressed in the serum of CRC patients with metastasis compared to those without metastasis. These results indicate that exosomal miR-92a-3p in patients’ plasma is important in predicting metastasis of CRC.

In conclusion, we provide evidence that CAFs can secret miR-92a-3p enriched exosomes into the tumor microenvironment. Exosomal miR-92a-3p promotes migration, invasion, metastasis, stemness, and 5-FU/L-OHP chemotherapy resistance by targeting FBXW7 and MOAP1 in CRC cells (Fig. 7h). Moreover, exosomal miR-92a-3p is up-regulated in the serum of CRC patients with metastasis and 5-FU/L-OHP chemotherapy resistance. We envision that blocking the function of exosomal miR-92a-3p secreted by CAFs could be used as an alternative modality for the prediction and treatment of CRC metastasis and therapy resistance.

## Additional file

Additional file 1: **Figure S1.** Isolation and characterization of matched CAFs and counterpart NFs from CRC patients. **Figure S2.** CAFs promote migration, invasion, and chemotherapy resistance in CRC. **Figure S3.** Isolation and characterization of exosomes. **Figure S4.** CAFs derived exosomes promote CRC cell proliferation and stemness. **Figure S5.** CAFs-exosomal miR-92a-3p promote aggressiveness and chemotherapy resistance in CRC. **Figure S6.** CAFs-exosomal miR-92a-3p promote stemness of CRC cells. **Figure S7.** FBXW7 and MOAP1 attenuate CAFs exosomal miR-92a-3p mediated promotion of CRC aggressiveness and drug resistance in vitro. **Figure S8.** FBXW7 and MOAP1 attenuate CAFs exosomal miR-92a mediated promotion of CRC aggressiveness and drug resistance in vivo. Supplemental materials and methods. **Table S1.** The primer sequences used in real-time PCR. (DOC 52500 kb)

## Abbreviations

3’UTR: 3′ untranslated region
5-FU: 5-fluoro-2,4(1 h, 3 h)pyrimidinedione
CAFs: cancer associated fibroblasts
CAFs-CM: CAFs derived conditioned medium
CAFs-exos: CAFs secreted exosomes
CRC: colorectal cancer
EMT: epithelial-mesenchymal transition
FAP: fibroblast activation protein
FSP-1: fibroblast specific protein 1
L-OHP: oxaliplatin
LSCM: laser scanning confocal microscope
NFs: normal fibroblasts
NFs-CM: NFs derived conditioned medium
NFs-exos: normal fibroblasts secreted exosomes
TEM: transmission electron microscopy
TME: tumor microenvironment
α-SMA: alpha-smooth muscle actin
