# Supplementary material for: CAFs secreted exosomes promote metastasis and chemotherapy resistance by enhancing cell stemness and epithelial-mesenchymal transition in colorectal cancer
Source: Mol Cancer. 2019 May 7;18:91. doi: 10.1186/s12943-019-1019-x (PMC6503554; doi:10.1186/s12943-019-1019-x)
Supplement: Supplementary file 1 — Figure S1. Isolation and characterization of matched CAFs and counterpart NFs from CRC patients. Figure S2. CAFs promote migration, invasion, and chemotherapy resistance in CRC. Figure S3. Isolation and characterization of exosomes. Figure S4. CAFs derived exosomes promote CRC cell proliferation and stemness. Figure S5. CAFs-exosomal miR-92a-3p promote aggressiveness and chemotherapy resistance in CRC. Figure S6. CAFs-exosomal miR-92a-3p promote stemness of CRC cells. Figure S7. FBXW7 and MOAP1 attenuate CAFs exosomal miR-92a-3p mediated promotion of CRC aggressiveness and drug resistance in vitro. Figure S8. FBXW7 and MOAP1 attenuate CAFs exosomal miR-92a mediated promotion of CRC aggressiveness and drug resistance in vivo. Supplemental materials and methods. Table S1. The primer sequences used in real-time PCR. (DOC 52500 kb) [file 12943_2019_1019_MOESM1_ESM.doc]

**Supplemental figures and figure legends**

**Figure S1**

**
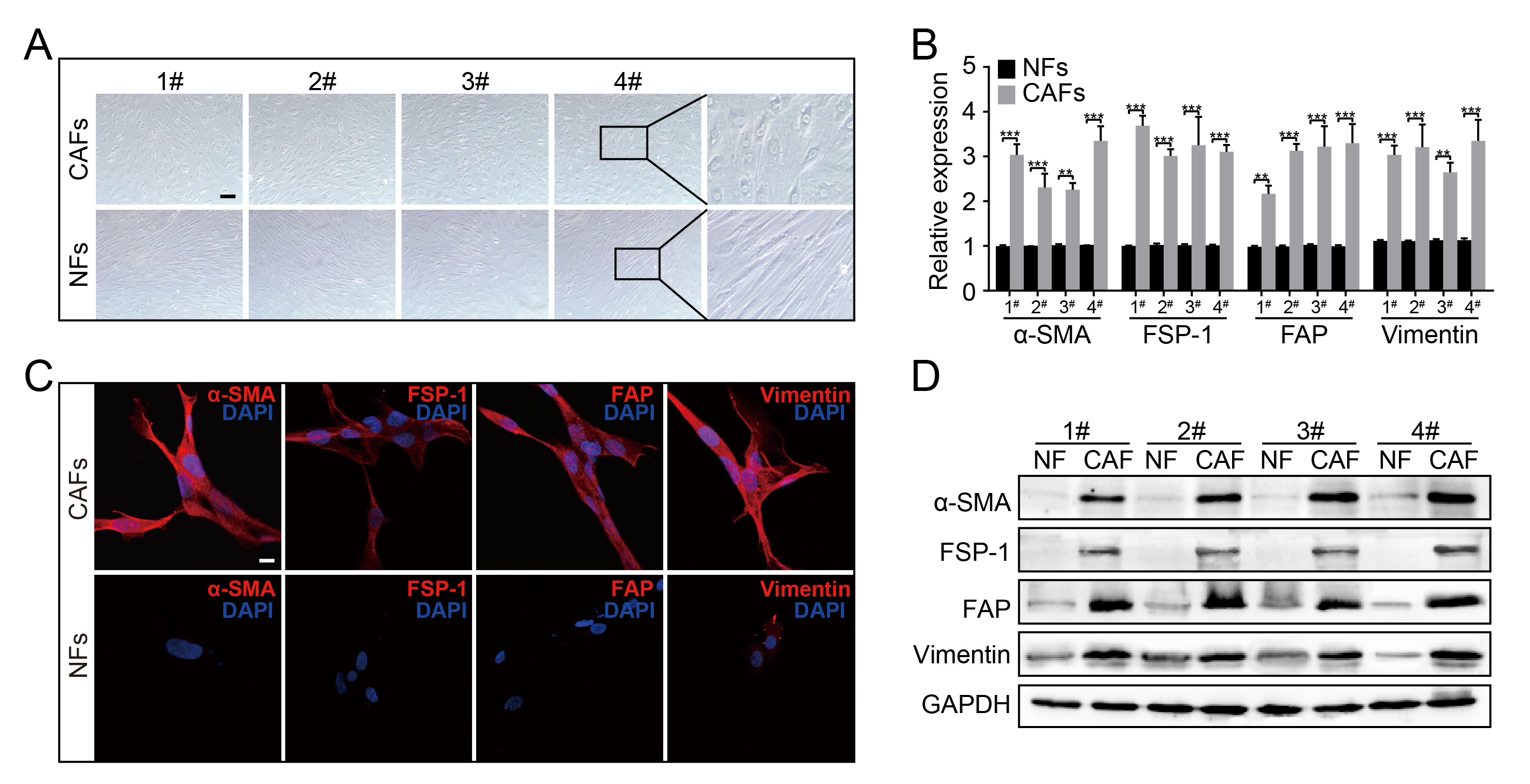
**

**Figure S1 Isolation and characterization of matched CAFs and counterpart NFs from CRC patients.**

(A) Microscopy observation of primary CAFs and NFs derived from CRC tissues and corresponding normal colorectal mucosa.

(B) Relative expression of α-SMA, Vimentin, FAP, and FSP-1 in NFs and CAFs by real-time PCR analysis.

(C-D) Expression of α-SMA，FSP-1, FAP, and vimentin in CAFs and NFs by immunofluorescence assay (C) and western blot (D).

**Figure S2**


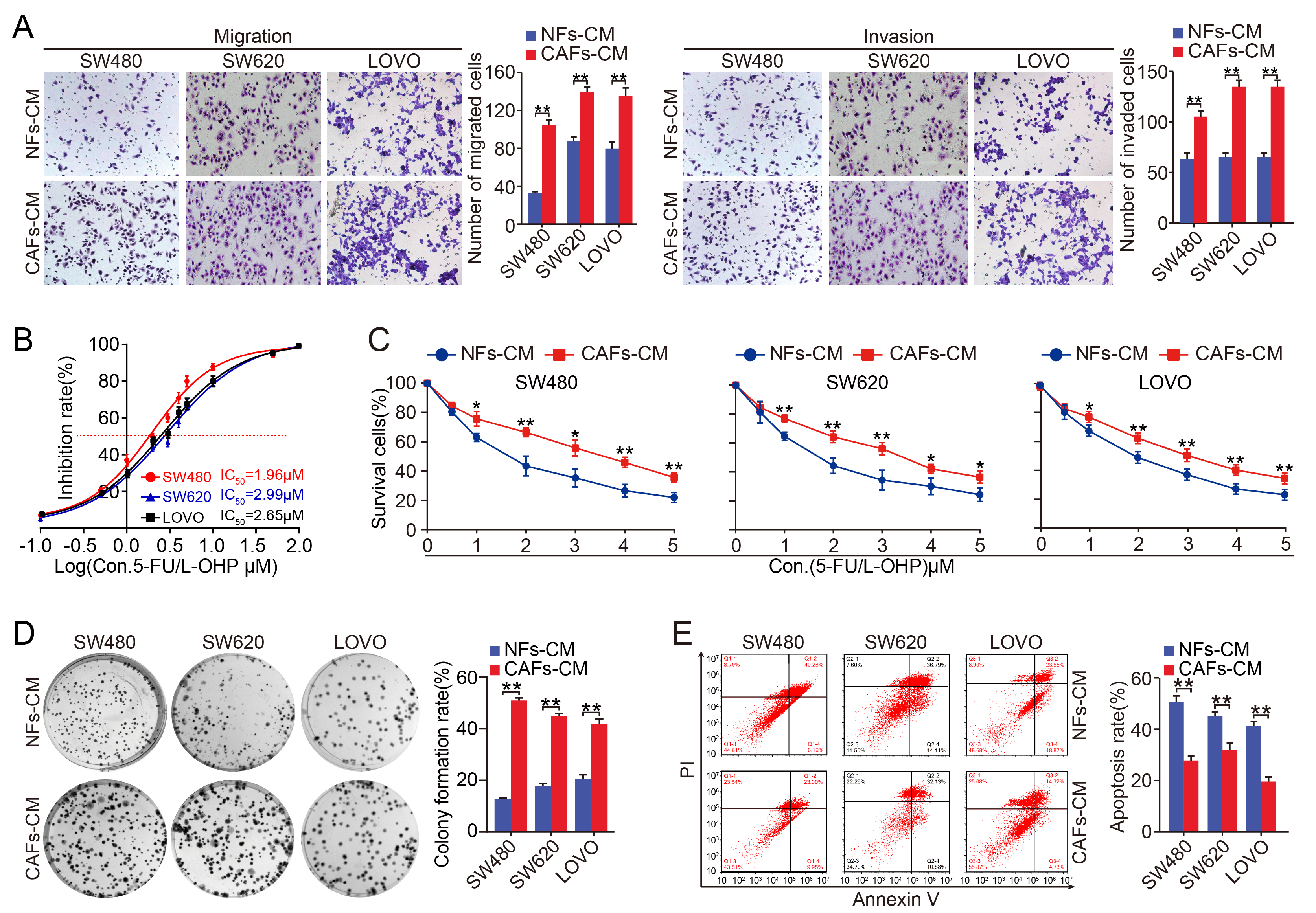


**Figure S2 CAFs promote migration, invasion, and chemotherapy resistance in CRC.**

(A) Effect of CAFs-CM and NFs-CM incubation on migration and invasion of SW480, SW620 and LOVO cells by Boyden chamber assay.

(B) IC_50_ curve of SW480, SW620 and LOVO cells under 5-FU/L-OHP therapy.

(C-E) Effect of NFs-CM and CAFs-CM on abilities of cell survival (C), colony formation (E) and apoptosis (E) of SW480, SW620 and LOVO cells by CCK-8, colony formation and flow cytometry assays.

**Figure S3**


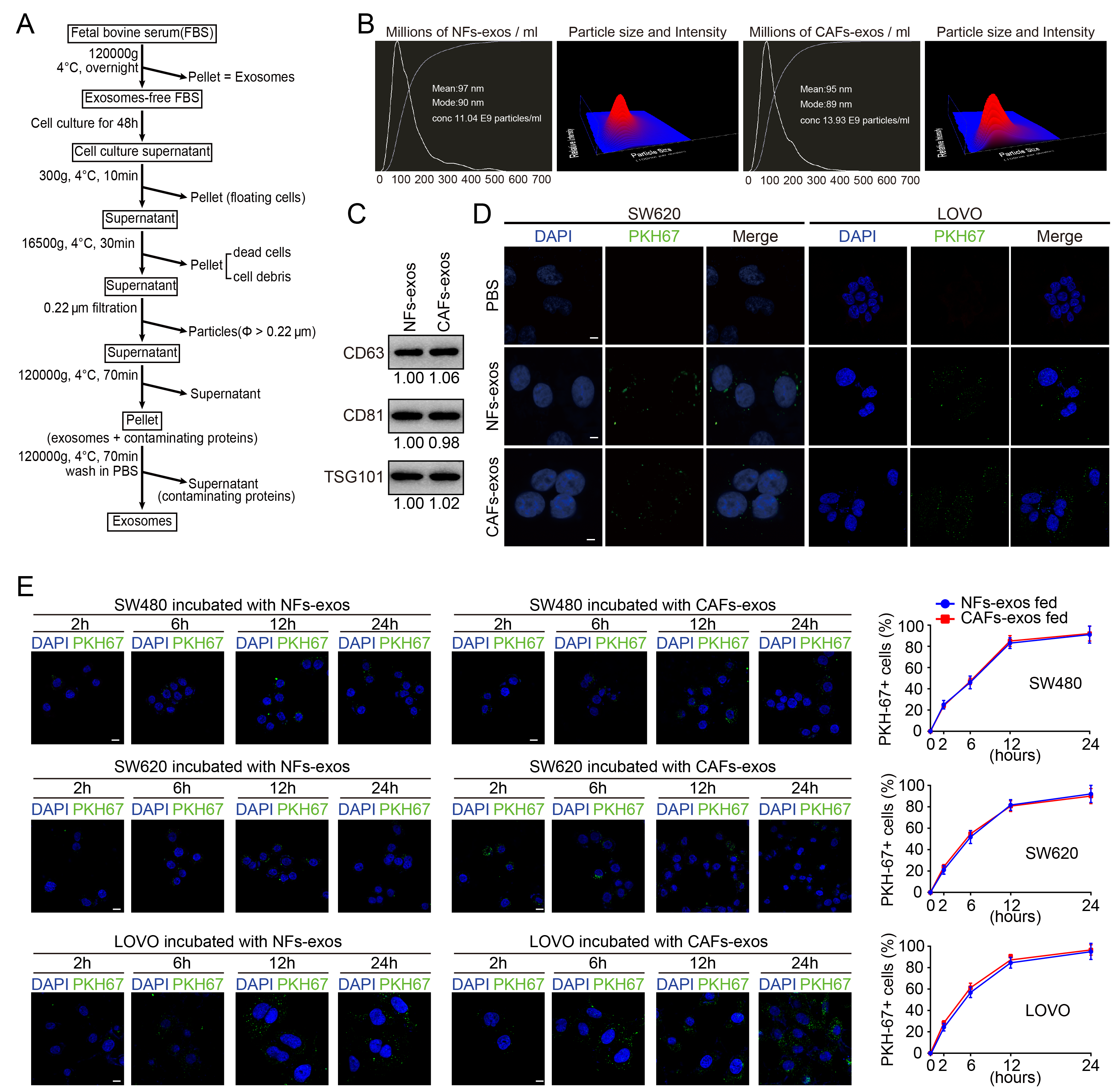


**Figure S3 Isolation and characterization of exosomes.**

(A) Protocols for the isolation of exosomes using ultracentrifugation method.

(B) Characterization of NFs-exos and CAFs-exos using Nanosight analysis.

(C) Expression of CD63, CD81 and TSG101 in NFs-exos and CAFs-exos derived from same amount of NFs and CAFs by western blot assay.

(D) Uptake of PHK67-labeled PBS, NFs-exos, and CAFs-exos by SW620 and LOVO cells observed by laser scanning confocal microscope.

(E) Uptake efficiency of PKH67-labeled NFs-exos and CAFs-exos by SW480, SW620 and LOVO cells observed by laser scanning confocal microscope.

**Figure S4**

**
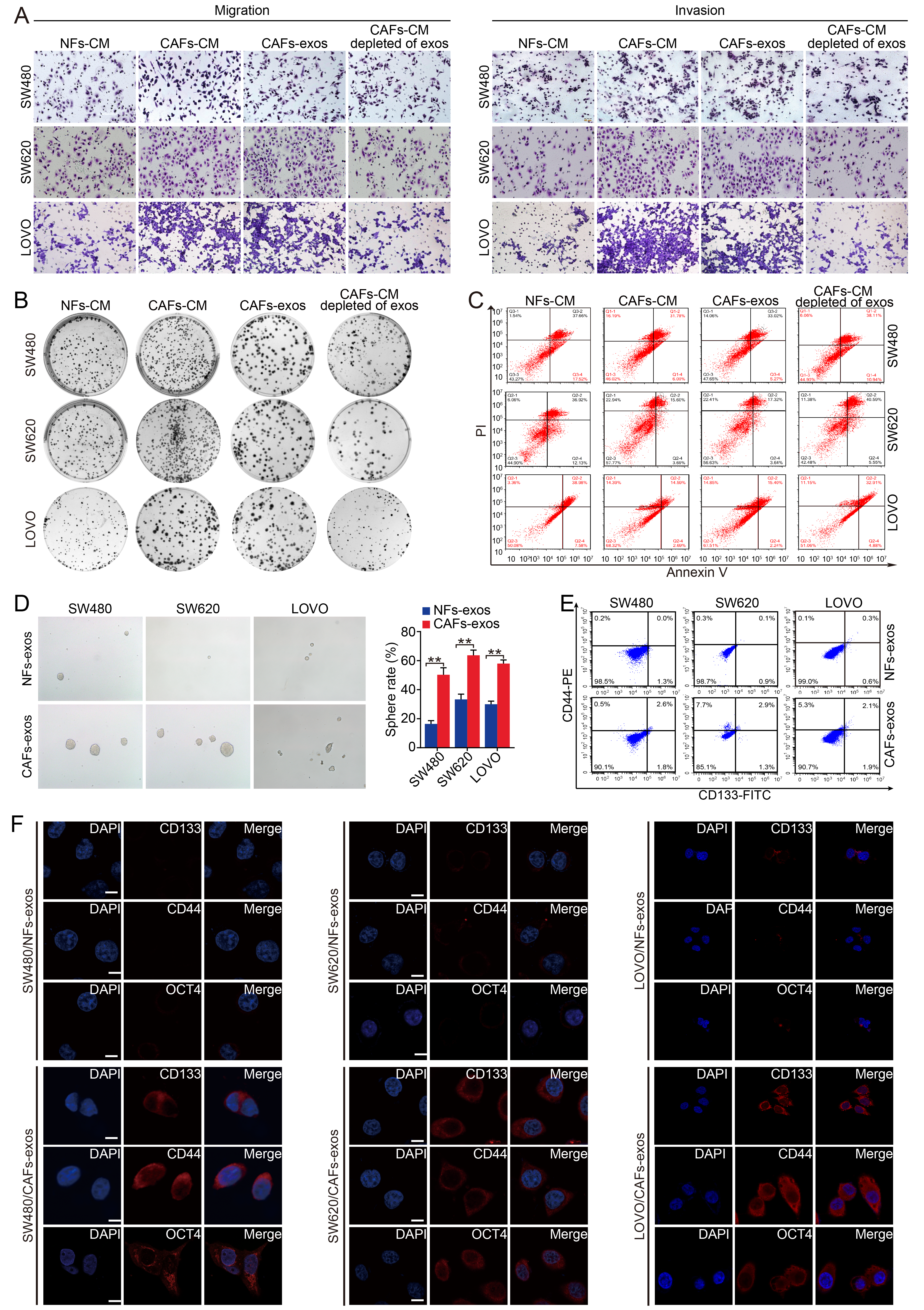
**

**Figure S4 CAFs derived exosomes promote CRC cell proliferation and stemness.**

(A) Effect of NFs-CM, CAFs-CM, CAFs-exos and CAFs-CM depleted of exosomes on migration and invasion of SW480, SW620 and LOVO cells.

(B&C) Effect of NFs-CM, CAFs-CM, CAFs-exos and CAFs-CM depleted of exosomes on colony formation abilities (B) and apoptosis (C) of CRC cells under 5-FU/L-OHP treatment.

(D-F) Effect of NFs-exos and CAFs-exos on sphere formation (D) and expression levels of stemness markers (E&F) in SW480, SW620 and LOVO cells.

**Figure S5**

**
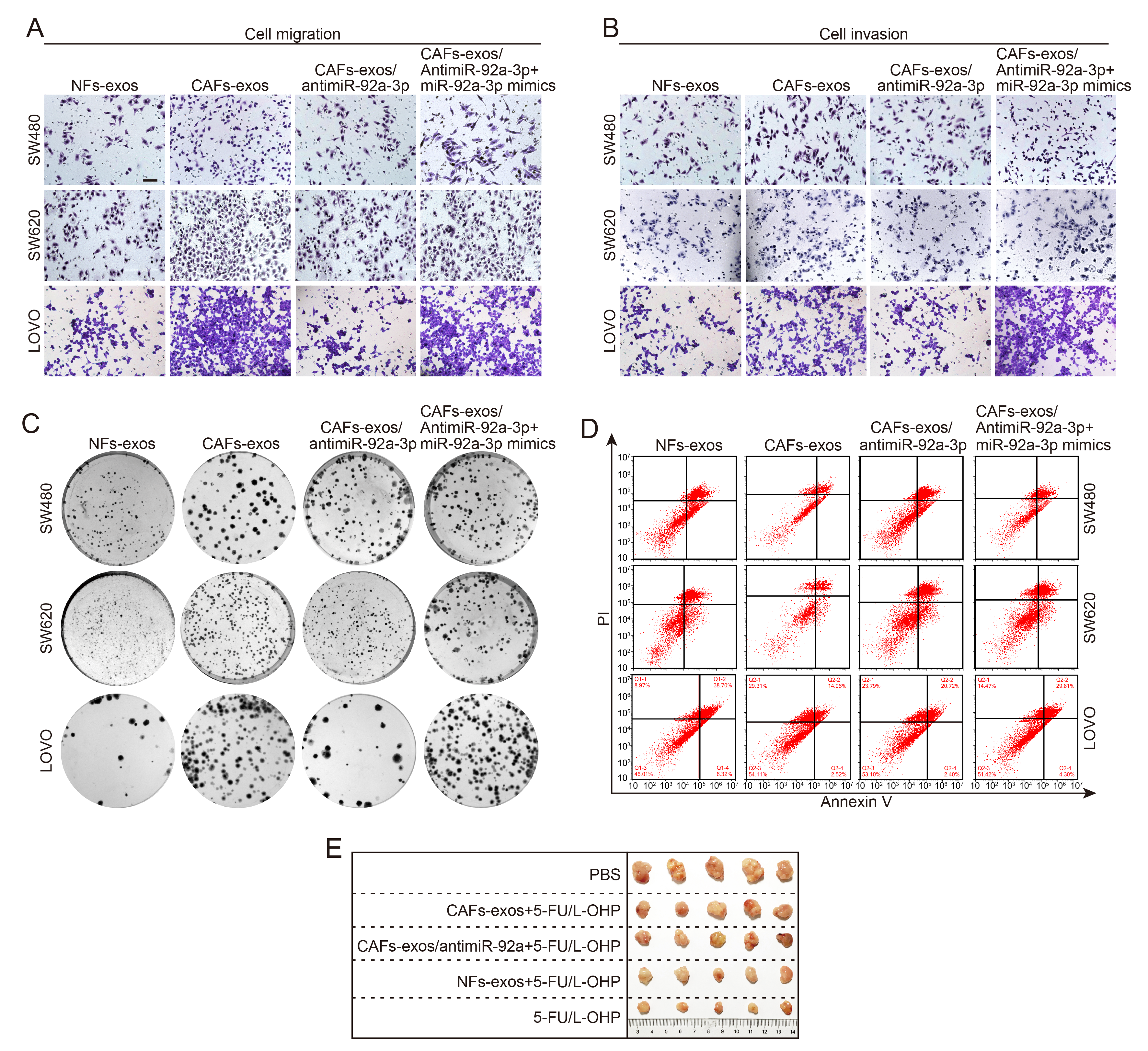
**

**Figure S5 CAFs-exosomal miR-92a-3p promote aggressiveness and chemotherapy resistance in CRC.**

(A&B) Effect of NFs-CM, CAFs-CM, CAFs-exos and CAFs-CM depleted of exosomes on cell migration (A) and invasion (B) abilities of SW480, SW620 and LOVO cells.

(C&D) Effect of NFs-CM, CAFs-CM, CAFs-exos and CAFs-CM depleted of exosomes on cell colony formation (C) and apoptosis (D) of CRC cells under 5-FU/L-OHP treatment.

(E) Effect of PBS, CAFs-exos+5-FU/L-OHP, CAFs-exos/antimiR-92a-3p, NFs-exos+5-FU/L-OHP, and 5-FU/L-OHP on cell proliferation in vivo.

**Figure S6**

**
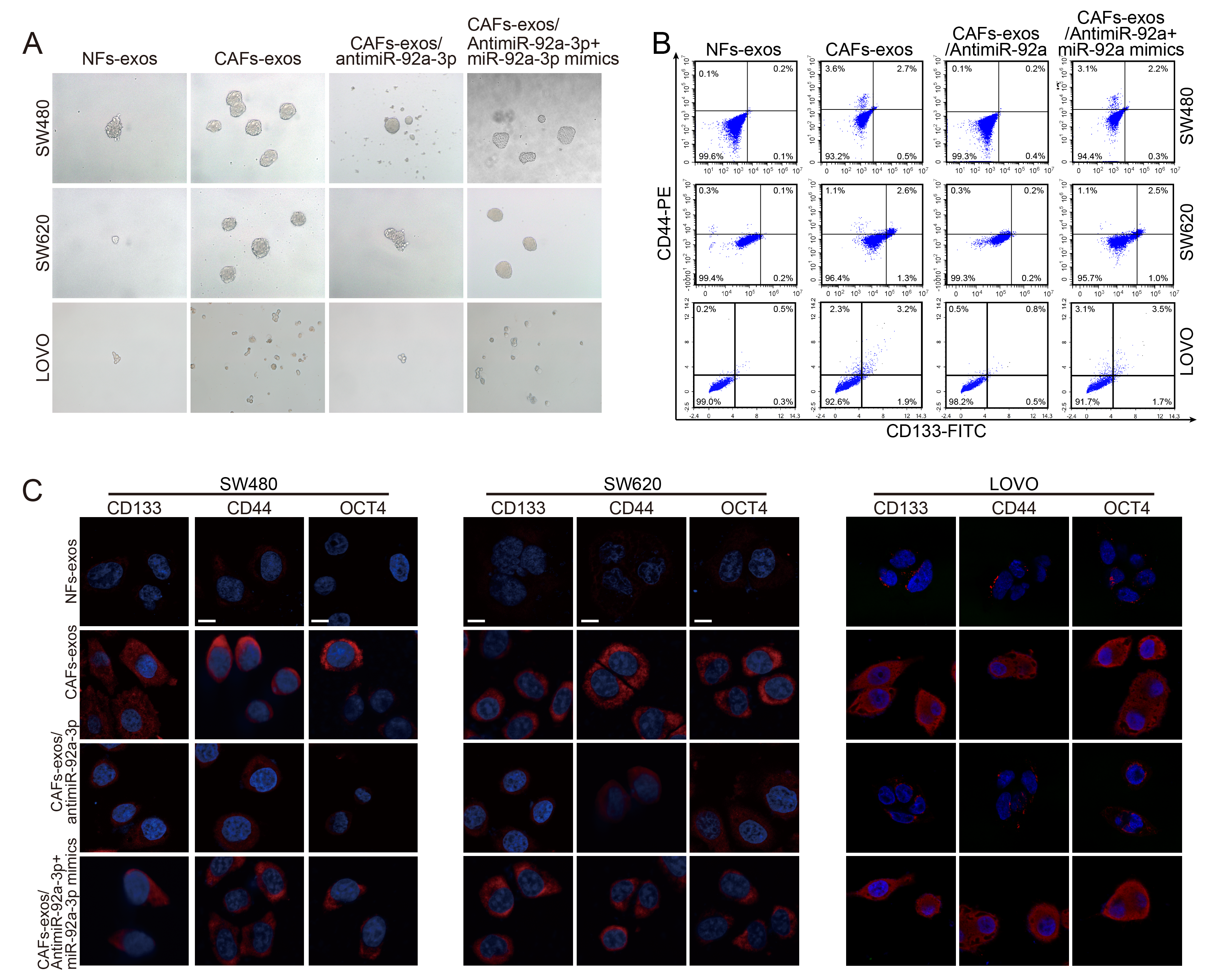
**

**Figure S6 CAFs-exosomal miR-92a-3p promote stemness of CRC cells.**

(A-C) Effect of NFs-exos, CAFs-exos, CAFs-exos/antimiR-92a-3p, CAFs-exos/antimiR-92a-3p+miR-92a-3p mimics on sphere formation (A) and expression levels of stemness markers (B&C) in SW480, SW620 and LOVO cells.

**Figure S7**

**
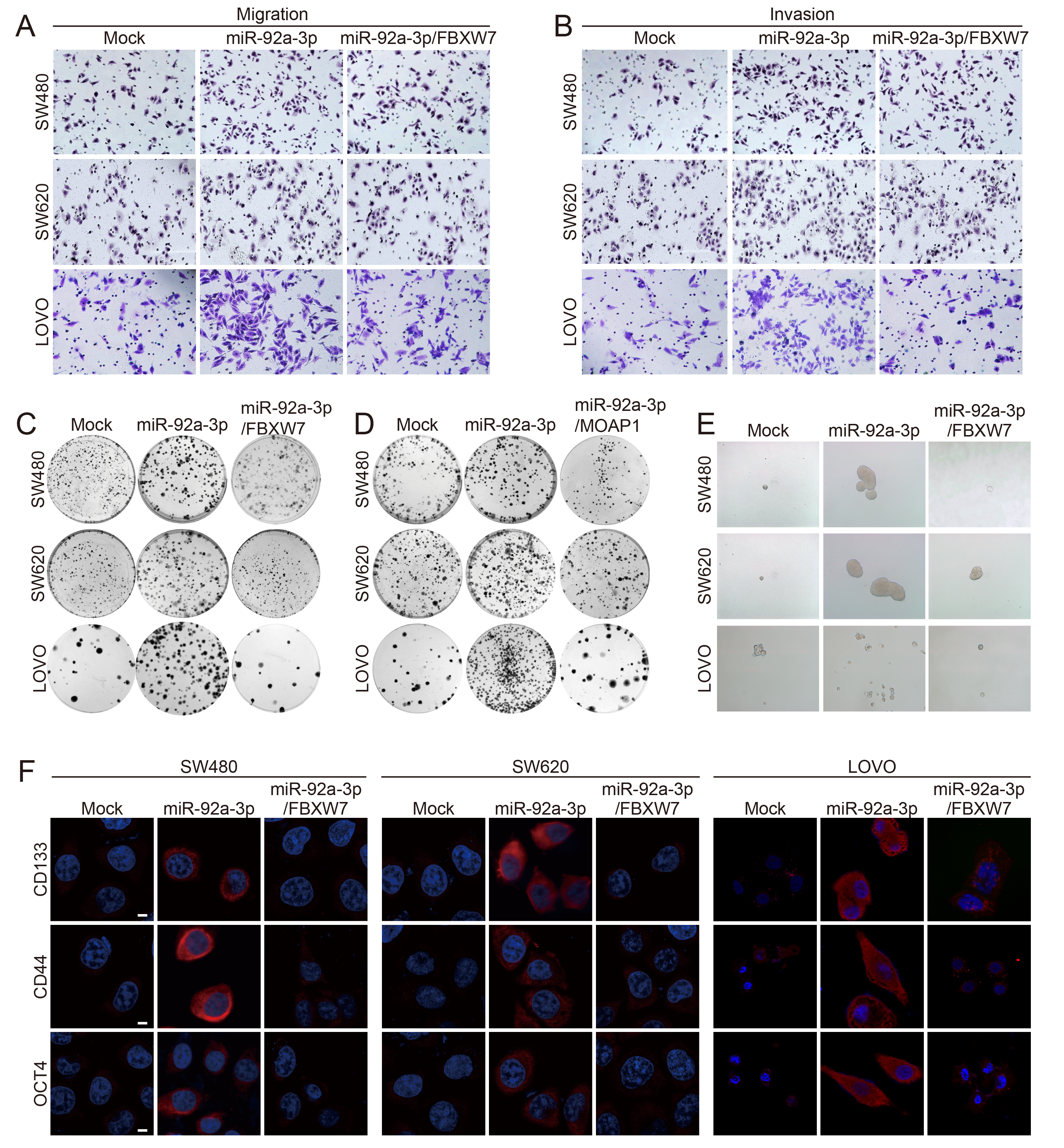
**

**Figure S7 FBXW7 and MOAP1 attenuate CAFs exosomal miR-92a-3p mediated promotion of CRC aggressiveness and drug resistance in vitro.**

(A-B) Effect of Mock, miR-92a-3p, and miR-92a-3p/FBXW7 transfection on invasion (A) and migration (B) of SW480, SW620 and LOVO cells by Boyden chamber.

(C-D) Effect of Mock, miR-92a-3p, miR-92a-3p/FBXW7, miR-92a-3p/MOAP1 transfection on colony formation abilities of SW480, SW620 and LOVO cells by plate colony formation assay.

(E) Effect of Mock, miR-92a-3p, miR-92a-3p/FBXW7 transfection on sphere formation of SW480, SW620 and LOVO cells by spheres formation assay.

(F) Effect of Mock, miR-92a-3p, miR-92a-3p/FBXW7 transfection on the expression of CD133, CD44 and OCT4 in SW480, SW620 and LOVO cells by immunofluorescence assay.

**Figure S8**

**
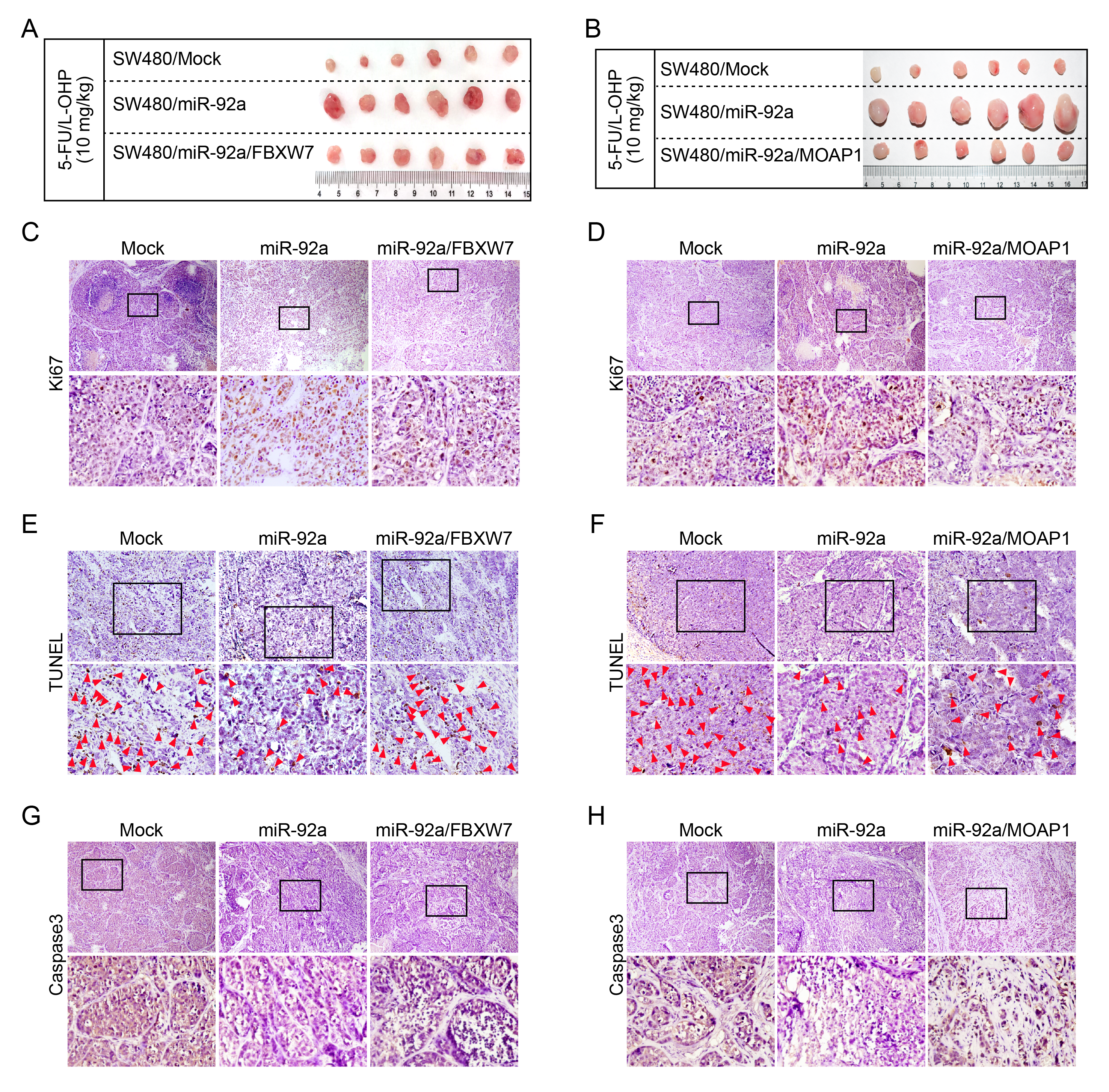
**

**Figure S8 FBXW7 and MOAP1 attenuate CAFs exosomal miR-92a mediated promotion of CRC aggressiveness and drug resistance in vivo.**

(A) The formation of subcutaneous tumors derived from SW480/Mock, SW480/miR-92a-3p, and SW480/miR-92a-3p/FBXW7 cells under 5-FU/L-OHP therapy by subcutaneous injection into the flank of mice (n = 6 in each group).

(B) The formation of subcutaneous tumors derived from SW480/Mock, SW480/miR-92a-3p, and SW480/miR-92a-3p/MOAP1 cells under 5-FU/L-OHP therapy by subcutaneous injection into the flank of mice (n = 6 in each group).

(C, E, G) Detection of proliferation and apoptosis by Ki-67 (C), TUNEL (E), and caspase3 (G) expression in tumor tissues derived from SW480/Mock, SW480/miR-92a-3p, and SW480/miR-92a-3p/FBXW7 cells by IHC and TUNEL assays.

(D, F, H) Detection of proliferation and apoptosis by Ki-67 (D), TUNEL (F), and caspase3 (H) expression in tumor tissues derived from SW480/Mock, SW480/miR-92a-3p, and SW480/miR-92a-3p/MOAP1 cells by IHC and TUNEL assays.

**Supplemental materials and methods**

**Human specimens and cell culture**

Tissues and serum of CRC patients were derived from patients at Department of General Surgery, Nanfang Hospital (Guangzhou, China). Informed consent was obtained to bank CRC tissues and serum for research purposes. The collection of CRC tissues and serum for research purposes was approved by the Ethics Committee of the Nanfang Hospital. CRC tissues were determined to be intratumoral by a pathologist and subsequently minced with a sterile blade and resuspended in a solution of DMEM with 20% fetal bovine serum (FBS, Gibco, Grand Island, USA), penicillin/streptomycin, amphotericin B and 3% collagenase for 2hrs at 37°C. Then samples were filtered through an 8μm mesh to remove undigested debris. The single cell suspension with viable fibroblasts was cultured in DMEM (10% FBS) for 2 to 3 weeks in a 24-well plate and then transferred to a T75 flask where it was continually maintained. Primary fibroblasts cultures were used for experiments up to passage ten. The counterpart fibroblasts were taken at >10 cm from the invasive edge of the tumor. SW480, SW620, HCT116, LOVO, HT29, and Ls174.t CRC cell lines were purchased from American Type Culture Collection and maintained in RPMI1640 containing 10% FBS at 37°C with 5% CO2.

**Isolation of exosomes from culture medium and serum samples**

To isolate exosomes from culture medium, CAFs or NFs were cultured in DMEM/F12 medium supplemented with 10% exosome-free FBS for 48 h, and culture supernatant was collected. Exosomes were isolated from the culture supernatant by differential ultracentrifugation. Briefly, culture supernatant was centrifuged at 300 g for 10 min and then at 165,00 g for 30 min at 4°C to remove cells, followed by filtration through a 0.22 μm filter (Millipore, USA) to remove particles. Exosomes were pelleted by ultracentrifugation at 120,000 g for 70 min. They were washed with sterilized PBS and purified by ultracentrifugation again at 120,000 g for 70 min. Exosomes were resuspended in PBS and filtered through 0.22 μm filters. The total protein concentration of isolated exosomes was determined by using a BCA protein assay kit (KeyGEN, China).

To isolate exosomes from serum samples of CRC patients, ExoQuick Exosome Precipitation Solution kit (System Biosciences, Mountain View, CA, USA) was used. Briefly, 500 μl of serum sample was mixed and incubated with 130 μl exosome precipitation solution for 30 minutes at 4°C. The ExoQuick/serum mixture was then centrifuged at 1,500× g for 30 minutes at room temperature. The pellet obtained was resuspended in 200 μl PBS.

**Characterization of exosomes**

The morphology of exosome was observed by transmission electron microscopy (FEI Tecnai 12, Philips, Netherlands). Briefly, exosomes were fixed with 4% paraformaldehyde and spotted onto glow-discharged copper grids. The copper grids were dried for 10 min at room temperature. Samples were stained with 2% uranyl acetate and dried for 10 min. Then samples were observed at 100 kV. Size distribution of exosomes was analyzed by measuring the rate of Brownian motion using a NanoSight LM20 system which was equipped with a fast video capture and particle‐tracking software (NanoSight, Amesbury, UK). Western blot analysis was performed to detecte exosome markers CD63, CD81, and TSG101.

**Exosomes internalization assays**

Cells (5 × 10^5^) were resuspended in 500 μl of exosomes-depleted cell media and incubated for the indicated time at 37°C with PKH-67 labeled-exosomes (2 μg) for confocal microscopy experiments. Samples were transferred to ice and washed 3 times with cold PBS (pH = 7.4). Cells were kept on ice and fluorescence measurements of live cells were taken by Leica TCS STED confocal microscope (Leica Microsystems).

**In vitro detection of miR-92a-3p transfer.**

For the transwell co-culture experiment, CAFs were grown on the 0.4 mm pore size transwell (Thermo Fisher Scientific) and transfected with 10nM green fluorescent 3’FAM-labelled miR-92a-3p oligo (Sigma-Aldrich Co.) for 24 h. Cells were then put together with colorectal cancer SW480 cells that had been grown on the cover slips in the bottom well of the transwell. After 24 h, the SW480 cells were fixed with 4% paraformaldehyde and observed under LSCM.

For the exosome treatment experiment, CAFs were transfected with 10nM green fluorescent FAM-labelled miR-92a-3p for 24 h. Cells were washed with PBS and incubated with medium containing exosome-free FBS for 48 h. Exosomes were isolated from CAF-CM using differential centrifugation. The pellet was suspended in serum-free medium and used to treat SW480 cells grown on cover slips. After 24 h, the SW480 cells were fixed with 4% paraformaldehyde and the nuclei were stained with 4,6-diamidino-2-phenylindole blue. The green signals in the SW480 cells were detected using LSCM.

**microRNA expression array**

The miRNA expression pattern among NF-derived exosomes, CAF-derived exosomes, SW480 cells incubated with NF-derived exosomes or CAF-derived exosomes analyzed using human Exiqon miRCURYTM LNA microRNAarray (Exiqon, Denmark). Three cell pellets were pooled in each group. The miRNA array experiment was carried out in KangChen Bio-tech Inc. (Shanghai, China).

**Western blot assay**

Immunoblotting was performed as follows: Proteins were extracted with a lysis buffer and then quantified by a bicinchoninic acid protein assay. Equivalent amounts of cell lysates were separated using SDS-PAGE and transferred to a polyvinylidene difluoride membrane (Roche Applied Sciences). Membranes were immunoblotted overnight at 4°C with antibodies, followed by the appropriate second antibodies. The bands were visualized using Pierce ECL Western Blotting Substrate (Thermo Fisher Scientific). Image density of the immunoblotting was determined by Gel densitometry (Bio-Rad).

**SiRNA and lentivirus transfection**

MiR-92a-3p-expressing lentivirus, miR-92a-3p-knockdown lentivirus, FBXW7-expressing lentivirus, MOAP1-expressing lentivirus and corresponding control lenti-vectors were all purchased from GeneCopoeia lnc. SW480 and SW620 cells were infected with indicated lentivirus or control lenti-vectors and were selected for further experiments by puromycin. For depletion of FBXW7, siRNA-FBXW7 sequences: 5’-CAAUUGUGUAGACGAUAUACU-3’; and a scramble siRNA sequence: 5’-ATACGCTATCGCTATCCGGTT-3’), which has no homology with the mammalian mRNA sequences, was used as control. Cells were transfected with siRNA using Lipofectamine®2000 according to the instructions (Invitrogen, Carlsbad, CA, USA).

**Immunohistochemistry staining**

Four-micrometer-thick histology sections from xenograft tumors and paraffin-embedded specimens were cut, deparafﬁnized using xylene, and hydrated through graded alcohol to water. Antigen retrieval was performed by boiling at 100°C for 10min in 10 mmol/L citrate buffers (pH = 6.0). Then sections were incubated with antibodies overnight at 4°C. Subsequently, the horseradish-peroxidase-conjugated anti-goat secondary antibody (DakoCytomation, Glostrup, Denmark) was applied and incubated for 1 hour at room temperature. The visualization signal was developed with 3, 3-diaminobenzidine tetra hydrochloride staining, and the slides were counterstained in hematoxylin.

**Immunofluorescence assay**

CRC cells were fixed in 4% paraformaldehyde for 10 min, blocked with phosphate-buffered saline (PBS) buffer containing 5% bovine serum albumin (BSA), and then incubated with antibodies at 4°C overnight, followed by incubation with fluorescein isothiocyanate (FITC)-conjugated secondary antibody and the nuclear counterstain diaminophenylindole (DAPI). After rinsing, the cells were analyzed using immunofluorescence microscopy.

**Sphere formation assay**

Briefly, SW480, SW620 and LOVO cells were incubated with CAFs-exosomes or NFs-exosmes for 48h and dissociated into single cells to 2 × 10^4^ cells/ml, and 100 μl of the cell solution was added into each well of a 96-well plate. Cells were cultured with DMEM/F12 medium (Gibco, USA) supplemented with 20ng/ml EGF (Gibco, USA), 10ng/ml Bfgf (Gibco, USA) and 10μl/ml B27 (Gibco, USA) for two weeks, with 50 μl of fresh mediu added at day 7. The number of sphere in each well ≥50 μm in diameter were counted under a microscope. Sphere formation rate for each well was the ratio of colony number to total cell number per well.

**Flow cytometry assay**

To determine cell apoptosis, Annexin V–fluorescein isothiocyanate/ propidium iodide staining (BD Biosciences, San Diego, CA) kit was performed to detect cell apoptosis. Briefly, SW480, SW620 and LOVO cells were seeded in six-well plates. After 48h of incubation with NFs-exos or CAFs-exos, the cells were collected using EDTA-free Tyrisin, washed twice with ice-cold PBS, and stained with Annexin V-FITC/ propidium iodide according to the manufacturer’s protocol. After staining for 15min, cells were detected with flow cytometry machine at an excitation wavelength of 488nm and an emission wavelength of 525nm.

To determine proportion of CD133/CD44 dual positive CRC cells, approximately 5 × 10^5^ cells were co-cultured with exosomes in 24-well plates for 24 h. Cells were washed three times with phosphate buffered saline (PBS), trypsinized, centrifuged (1500 rpm,5 min) and re-suspended in PBS (pH 7.4, with 2% FBS), and then stained with CD133-FITC, CD44-PE and isotype control IgG (BD Bioscience Pharmingen Inc., San Diego, CA, USA) for 30 min. Cells were then allowed to be detected by flow cytometry (BD LSRFortessa, San Diego, CA, USA).

**Luciferase activity assay**

For luciferase reporter assays, the putative miR-92a-3p complementary site in the 3’ untranslated region (3’ UTR) segment of FBXW7 and MOAP1 genes were amplified by PCR and inserted into vectors (RiboBio lnc.). Site-directed mutagenesis of the miR-92a-3p target site in FBXW7 and MOAP1 3’UTR was performed using the Quick Change Site-Directed Mutagenesis Kit (Stratagene). Co-transfections of FBXW7 3’ UTR-WT, FBXW7 3’ UTR-MUT, MOAP-1 3’ UTR-WT or MOAP-1 3’ UTR-MUT plasmid (RiboBio Inc.) with miR-92a-3p lentivirus vector into indicated cells were accomplished by using Lipofectamine 2000 (Invitrogen). Luciferase activity was measured 48 hours after transfection by the Dual-luciferase Reporter Assay System (Promega). Firefly luciferase signal was used for normalization. Each assay was repeated in three independent experiments.

**Animal models**

All animal experiments were conducted in accordance with the principles and procedures approved by the Committee on the Ethics of Animal Experiments of Southern Medical University. For 5-FU and L-OHP resistance model, 5 × 10^6^ SW480 cells were resuspended in PBS and subcutaneously injected into the flank of 4-week-old mice to establish xenografts. 5-FU/L-OHP (10mg / kg) or same volume of PBS every 3 days were injected subsequently. To study the role of CAFs-exos derived miR-92a-3p in 5-FU/L-OHP resistance, NFs-exos, CAFs-exos, and CAFs-exos/antimiR-92a-3p were injected into the vicinity of the subcutaneous tumors every 3 days. The tumor volume was calculated using the formula V = length × width^2^ / 2.

In mice lung metastasis model, 5 × 10^6^ SW480 cells with CM or SW480 cells with exosomes were co-injected into the tail veins of mice (n = 6 in each group). CM or exosomes (10μg) were then injected into tail veins every 3 days. Mice were sacrificed 6 weeks post injection. The number of metastatic nodules in lung were compared in each group. In mice liver metastasis model, 5 × 10^6^ SW620 cells were injected into the flank to form subcutaneous tumors. When the long axis reached 1 cm, tumors were resected and minced into 1mm^3^ tissues and transplanted to the ileocecal junction mucosa of mice (n = 8 in each group). To explore the role of CAFs-exos derived miR-92a-3p in metastasis, NFs-exos, CAFs-exos, CAFs-exos/antimiR-92a-3p (10μg) were injected to the ileocecal junction mucosa twice a week. The mice were sacrificed and bowels and livers were examined and collected for further analysis.

**Statistical analysis**

All statistical analyses were performed using SPSS 20.0 statistical software and were calculated from 3 independent experiments. Statistical significance was determined using Student’s t-test, Fisher’s exact test, or one-way analysis of variance (ANOVA) as appropriate. Growth curves were generated using ANOVA for repeated measurement. *P* < 0.05 was statistically significant.

Antibodies

| Antibody | Assay | Product code |
| --- | --- | --- |
| CD63 | WB | Ab59479, Abcam, USA |
| CD81 | WB | Ab79559, Abcam, USA |
| TSG101 | WB | Ab125011, Abcam, USA |
| BAX | WB | Ab32503, Abcam, USA |
| E-cadherin | WB | Ab15148, Abcam, USA |
| N-cadherin | WB | Ab18203, Abcam, USA |
| Caspase9 | WB | Ab52298, Abcam, USA |
| MOAP1 | WB | sc-271467, Santa Cruz, USA |
| GAPDH | WB | 10494-1-AP, Proteintech, USA |
| H2AX | WB | 10856-1-AP, Proteintech, USA |
| MMP7 | WB | 10374-2-AP, Proteintech, USA |
| MMP9 | WB | 10375-2-AP, Proteintech, USA |
| Ubiquitin | WB | 10201-2-AP, Proteintech, USA |
| β-catenin | WB, IF | Ab32572, Abcam, USA |
| Vimentin | WB, IF | 10366-1-AP, Proteintech, USA |
| α-SMA | WB, IF | 55135-1-AP, Proteintech, USA |
| FAP | WB, IF | 15384-1-AP, Proteintech, USA |
| FSP-1 | WB, IF | 16105-1-AP, Proteintech, USA |
| CD133 | WB, IF | 18470-1-AP, Proteintech, USA |
| OCT-4 | WB, IF | 11263-1-AP, Proteintech, USA |
| CD44 | WB, IF | 15675-1-AP, Proteintech, USA |
| Cytochrome C | WB, IF | Ab90529, Abcam, USA |
| Caspase 3 | WB, IHC | Ab13585, Abcam, USA |
| FBXW7 | WB, IHC | Ab109617, Abcam, USA |

Table S1. The primer sequences used in real-time PCR

| Gene | Primer sequence |
| --- | --- |
| miR-92a-3p | F: 5'-CACTTGTCCCGGCCTGTAAA -3' |
| miR-125b-5p | F: 5'-CGTCCCTGA-GACCCTAACTTGTGA-3' |
| miR-181d-5p | F: 5'- CCACCGGGGGAUGAAUGUCAC -3' |
| miR-185-5p | F:5'- GGGTGGAGAGAAAGGCAG-3' |
| miR-221-3p | F: 5'-AGCUACAUUGUCUGCUGGGUUUC -3' |
| miR-625-3p | F: 5'-GCGAGGGGGAAAGTTCTATAGT-3’ |
| FBXW7 | F: 5'-CGAACTCCAGTAGTATTGTGGACCT-3' |
|  | R: 5'-TTCTTTTCATTTTTGTTGTTTTTGTATAGA-3' |
| MOAP1 | F: 5'-GGGATGGACATGAACCCTCG -3' |
|  | R: 5'-CCTTCCAAGCAGTCTGTACTCC -3' |
| GAPDH | F: 5'-TGCACCACCAACTGCTTAGC-3' |
|  | R: 5'-GGCATGGACTGTGGTCATGAG -3' |
| E-cadherin | F: 5'-CGAGAGCTACACGTTCACGG-3' |
|  | R: 5'-GGGTGTCGAGGGAAAAATAGG -3' |
| N-cadherin | F: 5'-AGCCAACCTTAACTGAGGAGT-3' |
|  | R: 5'-GGCAAGTTGATTGGAGGGATG-3' |
| Vimentin | F: 5'-GACGCCATCAACACCGAGTT-3' |
|  | R: 5'-CTTTGTCGTTGGTTAGCTGGT -3' |
| CD133 | F: 5'-AGTCGGAAACTGGCAGATAGC-3' |
|  | R: 5'-GGTAGTGTTGTACTGGGCCAAT -3' |
| CD44 | F: 5'-CTGCCGCTTTGCAGGTGTA-3' |
|  | R: 5'-CATTGTGGGCAAGGTGCTATT -3' |
| OCT4 | F: 5'-CTGGGTTGATCCTCGGACCT-3' |
|  | R: 5'-CCATCGGAGTTGCTCTCCA -3' |
| α-SMA | F: 5'-AAAAGACAGCTACGTGGGTGA-3' |
|  | R: 5'-GCCATGTTCTATCGGGTACTTC -3' |
| FSP-1 | F: 5'-GATGAGCAACTTGGACAGCAA-3' |
|  | R: 5'-CTGGGCTGCTTATCTGGGAAG -3' |
| FAP | F: 5’-ATGAGCTTCCTCGTCCAATTCA-3’ |
|  | R: 5'-AGACCACCAGAGAGCATATTTTG-3' |
| CCND1 | F: 5'-AAAAGACAGCTACGTGGGTGA-3' |
|  | R:5’- CCTCCTTCTGCACACATTTGAA-3’ |
| C-MYC | F: 5'-GGCTCCTGGCAAAAGGTCA-3' |
|  | R:5’-CTGCGTAGTTGTGCTGATGT-3’ |
